# Release of *Staphylococcus aureus* extracellular vesicles and their application as a vaccine platform

**DOI:** 10.1038/s41467-018-03847-z

**Published:** 2018-04-11

**Authors:** Xiaogang Wang, Christopher D. Thompson, Christopher Weidenmaier, Jean C. Lee

**Affiliations:** 1 Division of Infectious Diseases, Department of Medicine, Brigham and Women’s Hospital and Harvard Medical School, 181 Longwood Avenue, Boston, MA 02115 USA; 2 Interfaculty Institute for Microbiology and Infection Medicine, University of Tuebingen, Elfriede Aulhorn Strasse 6, 72076 Tuebingen, Germany; 3 German Center for Infection Research, Partner Site Tuebingen, 72076 Tuebingen, Germany

## Abstract

Secretion of extracellular vesicles (EVs), a process common to eukaryotes, archae, and bacteria, represents a secretory pathway that allows cell-free intercellular communication. Microbial EVs package diverse proteins and influence the host-pathogen interaction, but the mechanisms underlying EV production in Gram-positive bacteria are poorly understood. Here we show that EVs purified from community-associated methicillin-resistant *Staphylococcus aureus* package cytosolic, surface, and secreted proteins, including cytolysins. Staphylococcal alpha-type phenol-soluble modulins promote EV biogenesis by disrupting the cytoplasmic membrane; whereas, peptidoglycan cross-linking and autolysin activity modulate EV production by altering the permeability of the cell wall. We demonstrate that EVs purified from a *S. aureus* mutant that is genetically engineered to express detoxified cytolysins are immunogenic in mice, elicit cytolysin-neutralizing antibodies, and protect the animals in a lethal sepsis model. Our study reveals mechanisms underlying *S. aureus* EV production and highlights the usefulness of EVs as a *S. aureus* vaccine platform.

## Abstract (web summary)

Extracellular vesicles (EVs) influence host-pathogen interactions, but EV biogenesis in gram-positive bacteria remains elusive. Here authors characterize EVs from *Staphylococcus aureus* and show that phenol-soluble modulins and autolysins promote EV biogenesis by disrupting the membrane and cell wall.

## Introduction

S*taphylococcus aureus* is a pathogenic bacterium that causes a wide spectrum of human infections, ranging from mild skin lesions to invasive, life-threatening infections. The pathogenesis of *S. aureus* infections is attributed to a wide array of virulence determinants, including surface proteins^1^ and glycopolymers^2^, as well as multiple secreted proteins, such as superantigens, leukotoxins, hemolysins, and proteases^3^. Although several specific export pathways have been described in *S. aureus*, the secretome often includes proteins that lack export signals and have typical cytoplasmic functions. The mechanisms by which cytoplasmic proteins are excreted by *S. aureus* have attracted recent interest^4,5^, and there is increasing evidence that these proteins may be secreted within extracellular membrane vesicles (EVs)^6–9^.

EVs are nano-sized, spherical, bilayered membrane vesicles with a cargo that includes diverse proteins, polysaccharides, nucleic acids, and lipids. EV formation by Gram-negative bacteria was first observed by electron microscopy in the 1960s^10^, and these bacteria secrete what are now referred to as outer membrane vesicles (OMVs). The generation of OMVs occurs by phospholipid accumulation in the outer leaflet of the outer membrane, followed by the formation of outer membrane protrusions that pinch off to form vesicles^11^. OMVs likely play important roles in bacterial pathogenesis due to packaging of multiple virulence factors^12^, and the ability of OMVs to serve as immune modulators by inducing innate and adaptive immune responses^13^.

Recent work has described the production and release of EVs from Gram-positive bacteria and fungi^6–9,14^. Because of the thick peptidoglycan (PGN) structure typical of Gram-positive microbes, EV biogenesis is a complex and poorly understood process. Toyofuku et al.^14^ recently reported that membrane vesicle formation in *Bacillus subtilis* was a result of prophage-encoded endolysins that generated holes in the PGN, facilitating EV release. EVs from Gram-positive organisms play important roles in host-pathogen interactions, as supported by reports that EVs contain biologically active toxins, exhibit cytotoxicity, and elicit proinflammatory mediators^9^. Moreover, toxin-positive *S. aureus* EVs elicit skin barrier disruption in mice with characteristic atopic dermatitis-like skin inflammation^15,16^. The toxicity of staphylococcal EVs has hampered a relevant study of their immunogenicity and potential use as a vaccine platform.

Despite repeated efforts to develop experimental vaccines and immunotherapeutics against *S. aureus*, neither have proven effective in preventing staphylococcal infections in humans^17^. Mice immunized with native *S. aureus* EVs produced a robust T-cell response and were protected against lung infection, but EV toxicity was not addressed in this study^18^. The development of EVs as a *S. aureus* vaccine platforms will require characterization of the mechanisms of EV biogenesis to enable consistent production with adequate quality assurance.

In this study, we generate EVs from a predominant community-acquired, methicillin-resistant *S. aureus* (CA-MRSA) clone in the United States. Our study reveals distinct mechanisms that facilitate EV production at multiple stages. Phenol-soluble modulins (PSMs) act at the membrane level to facilitate vesicle budding at the cytoplasmic membrane; whereas, cell wall porosity is modulated by PGN cross-linking and production of autolysins. Our results demonstrate the cytotoxicity of native *S. aureus* EVs for multiple cell types. By genetically engineering a non-toxic *S. aureus* mutant to over-produce detoxified cytolysins, we show that engineered EVs (eng-EVs) are immunogenic, non-toxic, and protect mice against *S. aureus* lethal sepsis. Our investigation describes a vaccine platform and provides the basis for further studies on the impact of EVs on the pathogenicity of *S. aureus* and other Gram-positive pathogens.

## Results

### Isolation of *S. aureus* EVs

**Fig. 1:**
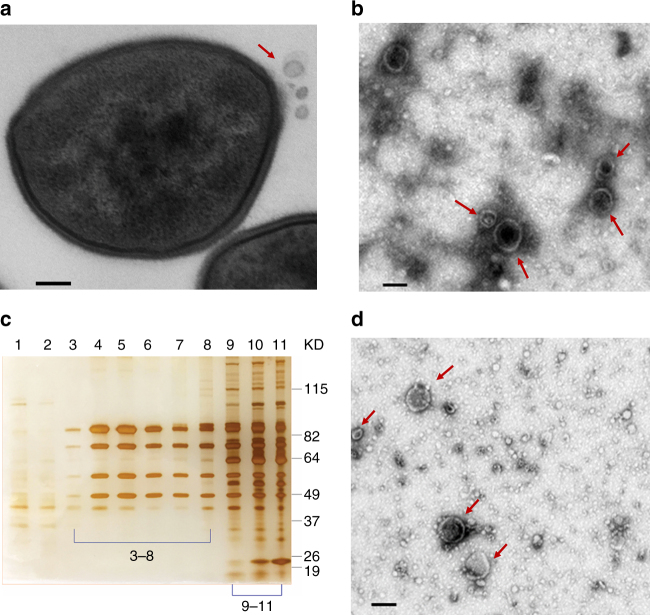
Extracellular vesicles from *S aureus* JE2. The bacteria were cultivated in TSB. **a** Ultrathin sections of *S. aureus* JE2 examined by TEM revealed EVs (indicated by a red arrow) released from the cell wall. **b** Crude EVs (red arrows) pelleted by ultracentrigation from the JE2 culture supernatant were imaged by TEM. **c** EVs were purified by density gradient ultracentrifugation (Optiprep), and fractions were visualized by silver-stained SDS-PAGE. **d** Fractions 3–8 were pooled; OptiPrep was removed by diafiltration, and the samples were imaged by TEM. EVs were not visualized in fractions 9–11. Scale bar, 100 nm

Ultrathin sections of JE2^19^ cells examined by transmission electron microscopy (TEM) revealed vesicle-like structures released from the *S*. *aureus* cell surface (Fig. 1a). We isolated the EVs by concentrating the culture supernatants to remove molecules <100 kDa before ultracentrifugation to pellet the EVs, shown in Fig. 1b. To remove non-membranous proteins, protein aggregates, and denatured EVs, Optiprep-based density gradient centrifugation was performed on the crude EV preparations. Consecutive Optiprep fractions (10 µl) were subjected to SDS-PAGE. Little silver-stained material was recovered from fractions 1 and 2 (Fig. 1c). Samples with similar protein banding patterns (fractions 3–8 and 9–11) were pooled, diafiltered, and examined by TEM. EVs were observed in fractions 3–8 (Fig. 1d), but not from fractions 9–11, indicating that EVs were distributed in fractions containing 20−35% Optiprep.

### Protein composition of *S. aureus* EVs

A proteomic analysis of purified JE2 EVs by liquid chromatography–tandem mass spectrometry (LC–MS/MS) identified 165 proteins (Supplementary Data 1), including alpha toxin (Hla), leukocidin subunits (LukS-PV, LukF-PV, LukE, LukD, HlgB, and HlgC), adhesins (ClfA, ClfB, SdrD, SdrE, Efb, and Ebh), MntC, proteases, and immune evasion factors (Sbi, phenol-soluble modulins, catalase, CHIPS, and SodA). Other proteins of interest included penicillin-binding proteins, autolysins (Atl, Sle1, and other putative autolysins with predicted N-acetylmuramoyl-l-alanine amidase activity), proteins involved in iron acquisition, and multiple other lipoproteins. Bioinformatic analyses revealed that 46% of EV proteins were cytoplasmic (*n* = 76), 16% were extracellular proteins (*n* = 27), 16% had an unknown localization (*n* = 27), 12% were membrane proteins (*n* = 19), and 10% were cell wall associated proteins (*n* = 16).

### Phenol-soluble modulins promote EV release

*S. aureus* secretes PSMs, which are a family of amphipathic, alpha-helical, surfactant-like peptides that are proinflammatory and show cytolytic activity against neutrophils, erythrocytes, epithelial cells, and endothelial cells^20,21^. Alpha-type PSMs are required for mobilizing lipoproteins from the staphylococcal cytoplasmic membrane, a process essential for activating TLR2^22^, as well as the export of cytoplasmic proteins, consistent with the membrane-damaging activity of PSMs^5^. Because the cargo of *S. aureus* EVs is enriched for both lipoproteins and cytoplasmic proteins, we evaluated whether PSM peptides were critical for EV generation.

**Fig. 2:**
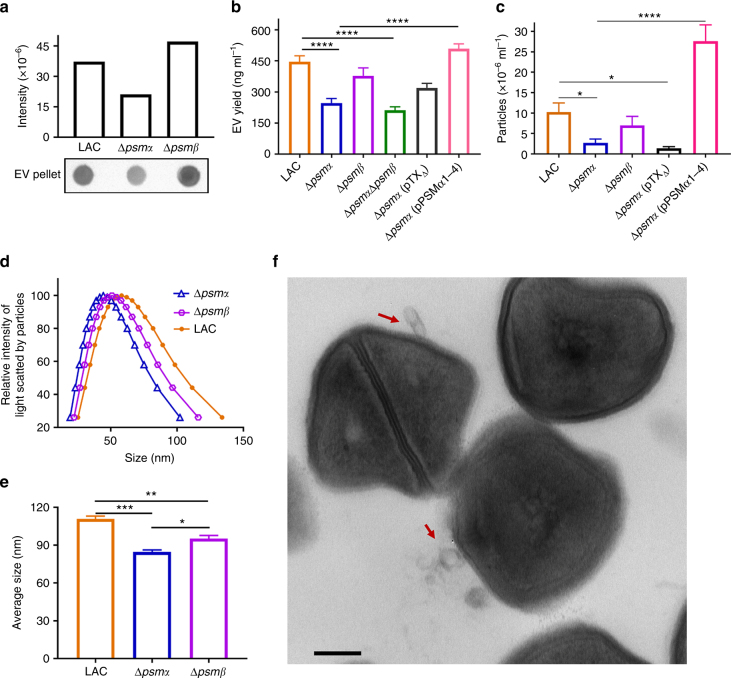
Alpha-type phenol-soluble modulin peptides promote *S. aureus* EV production. **a** EV production from strain LAC and its isogenic mutants lacking *psmα* or *psmβ* was evaluated by dot-blotting EV suspensions purified from the same volume of bacterial culture, **b** by quantification of total EV protein abundance, or **c** by direct EV quantification of EV particles with nanoparticle tracking analysis. **d** The size distribution and **e** average size of EVs purified from WT and ∆*psmα* and ∆*psmβ* mutants were analyzed by dynamic light scattering, and the data were generated with ZataPALS particle sizing software. Dot-blotting was repeated at least twice, and a representitative result is presented. Signal intensity quantified by Image Studio Lite software is shown above the blot. EV quantification by other methods was calculated from at least three independent experiments and expressed as mean ± s.e.m. **f** An electron micrograph of WT strain JE2 carrying pTX_∆_ expressing the genes encoding PSMα1-4 is shown. Scale bar, 200 nm. Samples were compared with a One-way ANOVA with Dunnett’s multiple comparison test (Fig. 2b, c) or with Tukey’s multiple comparison test (Fig. 2e). *P < 0.05, **P < 0.01, ***P < 0.001, ****P < 0.0001

We measured EV production by the WT USA300 LAC strain (the parent strain of JE2), as well as LAC ∆*psmα*, ∆*psmβ*, and ∆*psmα*∆*psmβ* mutants^21^. Dot immunoblot analysis revealed that deletion of *psmα* genes reduced EV production (Fig. 2a). Likewise, protein assays and nanoparticle tracking analysis (NTA) indicated that the *psmα* mutant showed significantly reduced *S. aureus* EV yield (Fig. 2b) and particle number (Fig. 2c), respectively. The ∆*psmα* and ∆*psmα*∆*psmβ* double mutant produced comparable levels of EVs (Fig. 2b), indicating that PSMα peptides play the dominant role in this phenotype. Complemention with pTX_∆_ expressing *PSMα1-4* genes, but not the pTX_∆_ vector alone, restored EV production to the ∆*psmα* mutant (Fig. 2b, c). Mutation of the *psmα* genes significantly reduced *S. aureus* EV size (Fig. 2c, d); whereas, the ∆*psmβ* mutant produced EVs of intermediate size compared to that of wild-type (WT) LAC. We transduced pTX_∆_PSM*α1-4*^21^ into strain JE2, and its EV yield (protein content) increased from 184 ± 12 to 650 ± 17 ng ml^−1^ (*n* = 3). Nonetheless, electron micrographs of JE2 (pTX_∆_PSM*α1-4*) showed intact bacterial cells producing abundant EVs (Fig. 2f). Significant differences in bacterial numbers recovered from JE2 cultures with or without pTX_∆_PSM*α1-4* were not observed (Supplementary Fig. 1a), indicating minimal impact of EV formation on bacterial viability.

### PGN cross-linking modulates EVs production

**Fig. 3:**
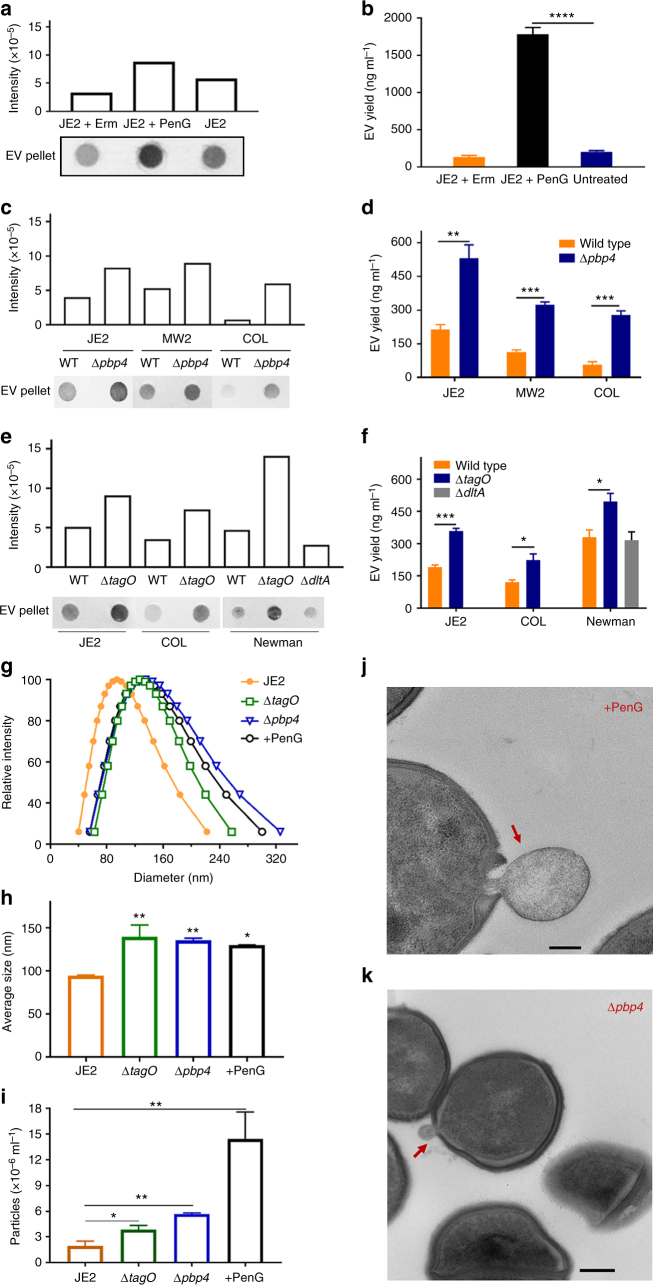
Reductions in peptidoglycan cross-linking increase *S. aureus* EV production and size. **a** Dot blots were performed on JE2 EVs treated with subinhibitory concentrations of penicillin G (PenG) or erythromycin (Em) and probed with mouse EV antiserum. **b** EV protein abundance was quantified and expressed as ng EV protein ml^−1^ culture. **c** EV production from *S. aureus* strains JE2, MW2, COL, or their isogenic penicillin-binding protein 4 (*pbp4)* mutants was evaluated by dot-blotting EV suspensions or **d** by quantification of total EV protein yield. **e** EV production from strains JE2, COL, Newman, and their ∆*tagO* (WTA-deficient) or ∆*dltA* (lacking alanine modifications on WTA) mutants was evaluated by dot-blotting EV suspensions or **f** by quantification of total protein yield. **g** The size distribution and **h** average size of EVs isolated from JE2, PenG treated JE2, and ∆*pbp4* and ∆*tagO* mutants were measured by dynamic light scattering. **i** EV particles from JE2, PenG treated JE2, and ∆*pbp4* and ∆*tagO* mutants were quantified by nanoparticle tracking analysis. Electron micrographs of **j** JE2 cells treated with PenG or **k** carrying a *pbp4* mutation are shown. Scale bar, 200 nm. Dot immunoblot assays were performed at least twice with similar results; a representative blot is shown. EV protein yield and EV particle quantification experiments were calculated from at least three independent experiments and expressed as mean ± s.e.m. The data were analyzed using one-way ANOVA with Dunnett’s multiple comparison test (Fig. 3b, h, i) or using Student’s *t*-test (Fig. 3d, f). *P < 0.05, **P < 0.01, ***P < 0.001, ****P < 0.0001

Unlike OMVs produced by Gram-negative microbes, *S. aureus* cytoplasmic membrane-derived EVs must traverse a PGN cell wall structure before cellular release. To determine whether the degree of PGN cross-linking affected *S. aureus* EV biogenesis, we cultured *S. aureus* JE2 in medium with a sublethal concentration (0.2 µg ml^−1^) of penicillin G (PenG) that has been shown to decrease PGN cross-linking^23^. Treatment with a sublethal concentration (0.1 µg ml^−1^) of erythromycin (Em) served as an antibiotic control that has no effect on PGN cross-linking. Compared to EVs recovered from untreated cultures or cultures incubated with Em, the EV yield from PenG-treated cultures was distinctly higher (Fig. 3a). When the EV protein content was quantified from a fixed volume of culture left untreated or treated with sublethal antibiotic concentrations, we observed a 10-fold increase in EV yield from PenG-treated cultures (Fig. 3b). EV production had little effect on bacterial viability since differences were not observed in bacterial numbers recovered from JE2 cultured with or without PenG (Supplementary Fig. 1b).

*S. aureus* penicillin-binding protein 4 (PBP4) is a carboxypeptidase that is essential for secondary cross-linking of PGN, and a *pbp4* mutant shows a significant reduction in PGN cross-linking^24^. As predicted, both dot blot (Fig. 3c) and EV protein yield assays (Fig. 3d) showed increased EV production by JE2Δ*pbp4*, and the protein yield was threefold higher than the wild-type JE2 strain. We also measured EV production in MRSA isolates MW2, COL, and their *∆pbp4* mutants; the relative increase in EV yield in the mutant strains (Fig. 3c, d) was consistent with that of JE2*∆pbp4*.

WTA is a PGN-anchored glycopolymer that is major component of the *S. aureus* cell wall and plays a critical role in cell wall homeostasis^2^. The *tagO* gene encodes an *N*-acetyl glucosamine-phosphate transferase enzyme that catalyzes the first step in WTA biosynthesis^25,26^, and deletion of *tagO* gene abrogates *S. aureus* WTA production^27^. Compared to the WT strains JE2, COL, and Newman, *tagO* mutants showed an enhanced signal in the dot immunoblot assay for EV production (Fig. 3e). Likewise, quantitative analysis of EV protein yield showed that all three *tagO* mutants produced significantly more EVs than the parental isolates (Fig. 3f). Thus, WTAs negatively modulate *S. aureus* EV production, consistent with reports showing that *tagO* mutants are characterized by diminished PGN cross-linking^28^. The WTA backbone is decorated with ester-linked D-ala residues, which confer a zwitterionic charge to the polymer^29^. As shown in panels **e** and **f** of Fig. 3, production and yield of EVs by the *ΔdltA* mutant were similar to that of the parental strain Newman.

To determine whether EV size was affected by reduced PGN cross-linking, the size distribution of purified EVs was measured by dynamic light scattering (DSL). Treatment of JE2 cultures with PenG or mutation of *pbp4* or *tagO* resulted in a significant increase in the size distribution of EVs (Fig. 3g), as well as an increased EV average size (Fig. 3h) compared to untreated WT EVs. Because enhanced EV production and yield associated with reduced PGN cross-linking might be a result of larger EVs that would carry an increased cargo load, we quantified EVs by nanoparticle tracking analysis. As shown in Fig. 3i, treatment of JE2 cultures with PenG or mutation of *pbp4* or *tagO* resulted in suspensions containing significantly greater numbers of EV particles per ml compared to untreated WT EVs. Electron micrographs of bacterial cells treated with PenG (Fig. 3j) or carrying a *pbp4* mutation (Fig. 3k) showed EVs being released or budding, respectively, from the cell membrane. Taken together, our data indicate that *S. aureus* EV production is inversely proportional to the degree of PGN cross-linking.

### Autolysin Sle1 promotes the release of EVs

**Fig. 4:**
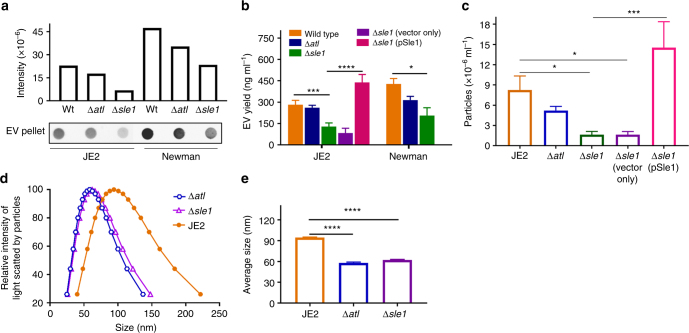
The autolysin Sle1 promotes EV biogenesis. **a** EV production from JE2 and different autolysis mutants (∆*atl* and ∆*sle1*) was evaluated by dot-blotting EV suspensions, **b** by quantification of total EV protein abundance, or **c** by EV quantification using nanoparticle tracking analysis. **d** The size distribution and **e** average size of EVs isolated from JE2 and the ∆*atl* and ∆*sle1* mutants were measured by dynamic light scattering. The dot immunoblot assay was repeated at least twice with similar results; a representative blot is shown. EV protein yield and EV particle quantification experiments were calculated from at least three independent experiments and expressed as mean ± s.e.m. The data were analyzed using one-way ANOVA with Dunnett’s multiple comparison test (Fig. 4b, c, e), For all panels, *P < 0.05, **P < 0.01, ***P < 0.001, ****P < 0.0001

Atl and Sle1 belong to a family of PGN hydrolases that plays a critical role in separation of daughter cells^30,31^, and Atl modulates the excretion of a subset of staphylococcal cytoplasmic proteins^4^. To determine whether PGN-hydrolases facilitate the release of EVs by altering the thick Gram-positive cell wall, we compared EV production from isogenic *atl* and *sle1* mutants with that of strains JE2 and Newman. Although both mutants showed reduced EV production (Fig. 4a), the reduction in yield was only significant in the *sle1* mutants (Fig. 4b). Likewise, NTA revealed that only the *sle1* mutant yielded a significantly lower EV concentration compared to WT JE2 (Fig. 4c). Complemention with pSle1 expressing the *sle1* gene, but not the pOS1-*hprK* vector alone, fully restored EV production to the JE2 ∆*sle1* mutant (Fig. 4b, c). Both *atl* and *sle1* mutants exhibited significantly reduced EV size compared to WT JE2 EVs (Fig. 4d, e).

Bacteriophages also produce PGN hydrolases called endolysins, which degrade the bacterial cell wall from within, resulting in cell lysis and release of progeny phages. Recently, Toyofuku et al.^14^ reported that prophage-encoded endolysins mediate the formation and release of EVs from *Bacillus subtilis* by generating a hole in the cell wall, leading to cell death. To investigate whether prophages or prophage-encoded endolysins are involved in *S. aureus* EV production, we analyzed *S. aureus* strains NCTC 8325 carrying ϕ11, ϕ12, and ϕ13 and 8325-4, which is cured of all three prophages^32^. Plating culture filtrates of NCTC 8325 on lawns of recipient strain RN4220 resulted in the formation of plaques, whereas culture filtrates of 8325-4 yielded no plaques (Supplementary Fig. 2a). EV yields and NTA revealed that NCTC 8325 and prophage-free strain 8325-4 produced comparable level of EVs (Supplementary Fig. 2b and 2c), indicating that prophage mobilization is not essential for the generation of *S. aureus* EVs.

### Effects of the *S. aureus* capsule on EV release

**Fig. 5:**
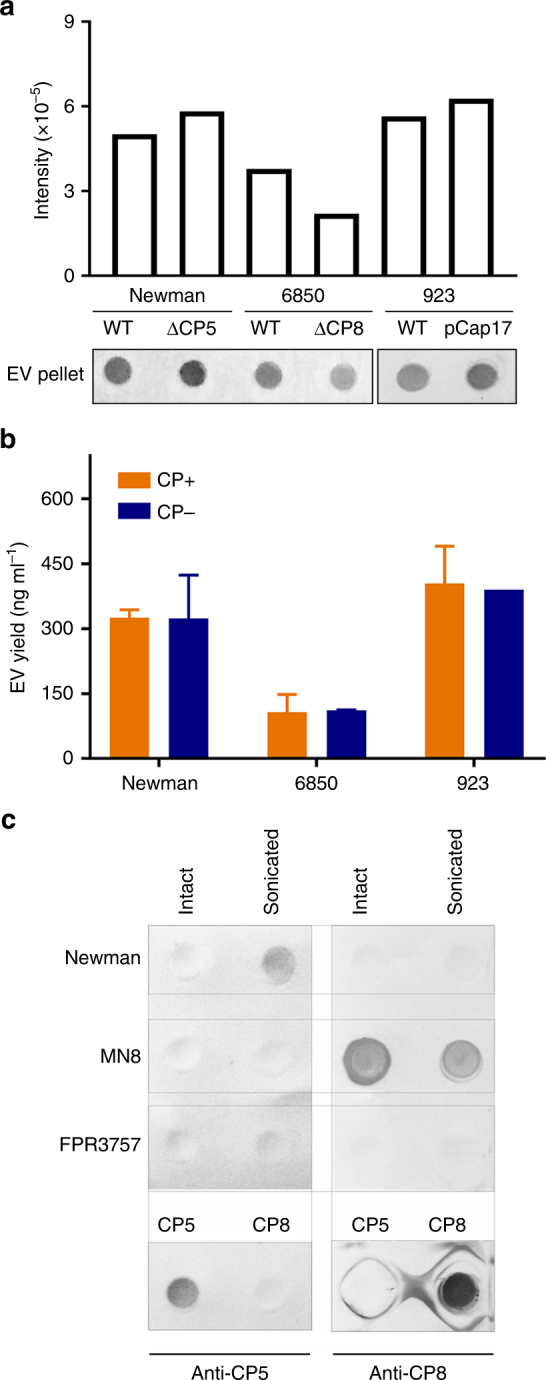
Effects of *S. aureus* capsular polysaccharide synthesis on EV production. **a** EV production from encapsulated *S. aureus* (Newman, 6850, 923 [pCP5]) or their CP-negative counterparts (Newman∆*cap5O*, 6850∆*capHIJK*, and 923) was evaluated by dot-blotting EV suspensions or **b** by quantification of total EV protein yield. **c** CP5 or CP8 was detected in intact or sonicated EVs (35 µg) from strains Newman, MN8, or FPR3757 by immunoblots probed with 1.2 µg ml^−1^ CP5-specific mAb 4C2 or CP8-specific mAb 5A6. Controls included 15 µg purified CP5 and CP8. The dot immunoblot assay was repeated at least twice with similar results; a representative blot is shown. EV protein yields were calculated from at least three independent experiments and expressed as mean ± s.e.m.

To determine whether the presence of capsular polysaccharide (CP) production impacted *S. aureus* EV biogenesis, we evaluated a number of isogenic CP+ and CP− strains. As shown in Fig. 5a, the CP phenotype had no obvious impact on the EV dot blot signal derived from WT or CP− mutants of strains Newman (CP5+) or 6850 (CP8+). Similarly, USA300 strain 923 (complemented to restore CP5 production) produced CP5^33^, but there was no effect on the EV signal levels achieved by dot blotting (Fig. 5a). Likewise, CP+ and isogenic CP− strains of Newman, 6850, and 923 produced comparable protein yields of EVs (Fig. 5b), indicating that CP did not modulate *S. aureus* EV production.

To investigate whether CP antigens were associated with *S. aureus* EVs, we performed CP immunoblots on EVs prepared from strains Newman (CP5+), MN8 (CP8+), and USA300 FPR3757 (CP−). CP antibodies react with surface-associated CP on intact EVs, whereas intravesicular CP would only be detected in sonicated EV preparations. Figure 5c shows that CP5 was only detected in sonicated, but not intact Newman EVs; whereas, CP8 was detected in both intact and sonicated MN8 EVs. EVs from CP− FPR3757 were non-reactive. Thus, both CP5 and CP8 were associated with EVs produced by CP+ *S. aureus*, although only CP8 was surface exposed.

### Detoxified EVs as a multicomponent vaccine platform

Multiple antigens were packaged within JE2 EVs, including lipoproteins, cytolytic toxins, surface proteins, and enzymes (Supplementary Data 1). If the toxicity of the EVs were eliminated, JE2 EVs could serve as a multicomponent *S. aureus* vaccine candidate. To repress the expression of cytolytic toxins, we mutated the *S. aureus* global regulator *agr* in strain JE2. We subsequently deleted *spa* (the gene encoding protein A) since an *agr* mutant overexpresses Spa, which binds to the Fcγ domain of immunoglobulin and dampens antibody development by cross-linking the Fab domain of V_H_3-type B-cell receptors^34^. The JE2 *agr* mutation significantly inhibited mRNA expression of *hla* (encoding alpha toxin) and the genes encoding all nine leukocidin subunits (Supplementary Fig. 3a). The JE2∆*agr*∆*spa* double mutant served as our *S. aureus* EV vaccine producing host strain. EVs from JE2, but not the JE2∆*agr*∆*spa* mutant, contained native Hla and LukE (Supplementary Fig. 3b) as assessed by western blotting. When we analyzed the protein content of JE2∆*agr*∆*spa* EVs by LC–MS/MS, many of the extracellular proteins present in JE2 WT EVs were not detected in JE2∆*agr*∆*spa* EVs. However, some antigens such as MntC and FhuD2 that protect mice against experimental *S. aureus* infections^35,36^ were present in EVs from the mutant strain. Neither protein A nor the toxins Hla, Panton-Valentine leukocidin (Luk-PVL), LukED, HlgCB, SelX, or PSMs were detectable in EVs purified from the JE2∆*agr*∆*spa* mutant (Supplementary Data 2). Although LukAB was still present in EVs from JE2∆*agr*∆*spa*, there was ≥86% reduction in the number of peptides detected in the mutant strain (Supplementary Data 1 and 2). Moreover, as indicated below, EVs recovered from the mutant strain showed no residual toxicity toward human leukocytes.

We immunized mice with 5 µg EVs from JE2∆*agr* or JE2∆*agr*∆*spa* mutants; control mice were given phosphate buffered saline (PBS). EVs from both mutants elicited a serum antibody response against sonicated WT EVs, although the antibody level elicited by ∆*agr* EVs was higher than that elicited by ∆*agr*∆*spa* EVs (Supplementary Fig. 4a). To examine the antigen profiles from EVs that elicited antibody responses after immunization, a bacterial lysate from USA300 strain FPR3757 was subjected to SDS-PAGE and immunoblotted with sera pooled from mice immunized with either ∆*agr* EVs or ∆*agr*∆*spa* EVs. Sera from ∆*agr∆spa* EVs-immunized mice reacted with more bacterial antigens than sera from ∆*agr* EVs-immunized mice (Supplementary Fig. 4b), suggesting that *∆agr∆spa* EVs elicited a greater diversity of antibodies than *∆agr* EVs. The immunized mice were then challenged with strain FPR3757, a heterologous USA300 isolate. Immunization of mice with EVs from JE2*∆agr∆spa*, but not EVs from JE2*∆agr*, provided significant protection against lethal sepsis (Supplementary Fig. 4c). Immunization with higher doses of JE2∆*agr∆spa* EVs mixed with alum did not significantly enhance immunogenicity (Supplementary Fig. 4d).

### Engineered EVs protect mice against lethal sepsis

To enhance the protective efficacy of detoxified EVs from JE2*∆agr∆spa*, we engineered JE2 to package non-toxic Hla_H35L_^37^ and the LukE monomer within eng-EVs. LukED, detected in 82% of blood isolates and 61% of nasal isolates^38^, targets human and murine neutrophils, macrophages, T cells, dendritic cells, NK cells, and erythrocytes^39^.

We expressed non-toxic Hla_H35L_ and LukE in strain JE2*∆agr∆spa* under control of the *spa* promoter, which is enhanced in an *∆agr* genetic background^40^. Thus, mRNA levels of Hla_H35L_ and LukE expressed in JE2*∆agr∆spa* were dramatically increased compared to expression in JE2*∆agr∆*spa or JE2*∆agr∆*spa with the empty vector (Supplementary Fig. 3c). Both Hla_H35L_ and LukE were detected by western blot in eng-EVs isolated from recombinant strain JE2*∆agr∆spa* (pHla_H35L_-LukE) (Supplementary Fig. 3b).

The relative toxicity of EVs prepared from WT strain JE2 and JE2*∆agr∆spa* vs. eng-EVs from JE2*∆agr∆spa* (pHla_H35L_-LukE) was assessed by incubating EVs in vitro with three different cell types. A549 cells are susceptible to Hla-mediated cytolysis, and WT strain JE2 EVs were toxic for A549 cells at concentrations as low as 1 µg ml^−1^. In contrast, JE2*∆agr∆spa* mutant EVs and the eng-EVs from JE2*∆agr∆spa* (pHla_H35L_-LukE) exhibited negligible toxicity (Supplementary Fig. 5a). HL60 cells are susceptible to the cytolysis induced by *S. aureus* leukocidins, and JE2 EVs, but not ∆*agr∆spa* or eng-EVs, were cytolytic for HL60 cells (Supplementary Fig. 5b**)**. Rabbit erythrocytes are susceptible to Hla, PSMs, and the leukocidins HlgAB and LukED^39^. JE2 EVs exhibited significant hemolytic activity, whereas no hemolysis resulted from ∆*agr∆spa* mutant EVs or eng-EVs (Supplementary Fig. 5c). These data demonstrate that the eng-EVs were non-toxic in vitro for mammalian cells.

**Fig. 6:**
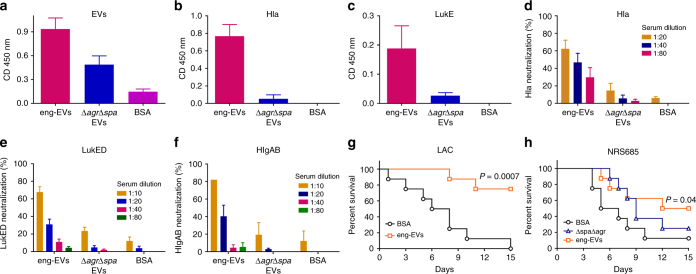
Immunogenicity and protective efficacy in mice of engineered-EVs. Antibody levels in sera (diluted 1:100) from mice immunized with eng-EVs were analyzed on ELISA plates coated with **a** JE2 sonicated EVs, **b** Hla, or **c** LukE. Data were expressed as mean ± s.e.m. The neutralizing activity of sera from mice immunized with BSA or different EV preparations was determined by either incubating serial dilutions of sera with **d** Hla, **e** LukED, or **f**, or HlgAB for 1 h at 37 °C before adding target cells. Control cells were incubated with toxins but no sera. Data are expressed as percent neutralization ± s.e.m. Mice (*n* = 8) immunized with different JE2 EV preparations were challenged IV with 8 × 10^7^ CFU *S. aureus* LAC (**g**) or 2 × 10^8^ NRS685 (**h**). Survival (comparing EV-immunized mice vs. BSA-immunized mice) was analyzed with the log-rank test

We immunized mice on days 0, 14, and 28 with 5 µg eng-EVs from JE2∆*agr∆spa* (pHla_H35L_-LukE) or with 5 µg EVs from the JE2∆*agr∆spa* mutant; control mice received 5 µg bovine serum albumin (BSA). Whereas sera from mice immunized with both eng-EVs and *∆agr∆spa* EVs, but not BSA, reacted by ELISA with sonicated WT JE2 EVs (Fig. 6a), only mice given the eng-EVs responded with antibodies to purified Hla (Fig. 6b) or LukE (Fig. 6c). These data indicate that recombinant proteins packaged within *S. aureus* EV are immunogenic.

To examine whether the antibodies elicited by the eng-EV vaccine were functional, toxin neutralizing assays were performed. Sera from mice immunized with eng-EVs effectively neutralized Hla at dilutions ranging from 1:20 to 1:80 (Fig. 6d), whereas serum neutralizing antibodies were low or undetectable in the BSA or ∆*agr∆spa* EV groups. Similarly, sera from mice immunized with eng-EVs, but not BSA or ∆*agr∆spa* EVs, were able to effectively neutralize LukED at dilutions ranging from 1:10 to 1:20 (Fig. 6e). Sera from mice immunized with eng-EVs also neutralized leukocidin HlgAB (Fig. 6f), but not PVL-SF or HlgCB leukotoxins.

The immunized mice were challenged with USA300 strain LAC or USA500 strain NRS685, a PVL-negative MRSA bacteremia isolate. We chose the latter strain because the PVL-S and PVL-F subunits can interact with LukE and LukD to form inactive hybrid complexes, which have been shown to influence LukED-mediated *S. aureus* virulence in mice^41^. As shown in Fig. 6g, h, immunization with eng-EVs provided significant protection against both *S. aureus* isolates in the lethal murine sepsis model. JE2∆*agr∆spa* EVs were not protective against the USA500 strain (Fig. 6h).

## Discussion

The production of membrane vesicles represents a secretory pathway common to mammalian cells, fungi, and bacteria that allows cell-free intercellular communication^42–44^. Microbial EVs encapsulate cargo that include lipids, proteins, glycans, and nucleic acids, which have been shown to play roles in microbial physiology, pathogenesis, and the transmission of biological signals into host cells to modulate biological processes and host innate immune responses^42,43,45,46^. In Gram-negative bacteria, EVs are generated by pinching off the outer membrane, but the mechanism(s) by which EVs escape the thick cell walls of Gram-positive bacteria, mycobacteria, and fungi are unknown. Once shed, *S. aureus* EVs can undergo cholesterol-dependent fusion with host cell membranes to deliver their toxic cargo^47^. *S. aureus* EVs are produced in vivo during experimental pneumonia in mice^47^. In this report, we demonstrate unique properties associated with EV production by JE2, a *S. aureus* USA300 strain representative of the prevalent CA-MRSA clone in the US. Similar to EVs characterized from other *S. aureus* isolates^47–49^, JE2 EVs encapsulate an array of bacterial antigens, including lipoproteins, exotoxins, and cytoplasmic proteins.

In this report, we evaluated putative factors that modulate the membrane and PGN related steps of EV release. PSMs are a group of small alpha helical peptides that have surfactant-like properties and potent cytolytic activity for leukocytes, epithelial cells, and endothelial cells^20^. PSMα peptides are 20–22 amino acids in length; whereas, PSMβ peptides are 43–45 amino acids in length. In our studies, PSMα peptides, but not PSMβ peptides, supported the generation of EVs from *S. aureus*. EVs from the PSMα mutant were less abundant and smaller in size compared with WT EVs. Chatterjee et al.^50^ reported that an *S. aureus* mutant that lacks the PSM transporter protein accumulates PSMs intracellularly, causing cytoplasmic membrane perturbations. Surfactants or surfactant-like proteins with amphipathic helical structures have been shown to insert into lipid monolayers and generate local deformation^51,52^. PSMs, due to their surfactant-like activity, as well as amphipathic helical structure, may enhance membrane curvature under cytoplasmic turgor pressure, resulting in membrane disruption and the formation of EVs. Although EVs from Gram-negative bacteria arise from the outer membrane rather than the plasma membrane, the biogenesis of OMV production is also thought to be due to perturbations in the outer leaflet of the membrane due to specific phospholipid accumulation therein^11^. Recently, Ebner et al.^5^ reported that *S. aureus* PSMα peptides-induced the cellular release of cytoplasmic proteins, lipids, nucleic acids, and ATP into culture supernatants, and that this effect was mediated by the membrane-damaging activity of the PSMα peptides. Because PSMα peptides promote EV production, and EVs encapsulate cytoplasmic proteins, lipids, and nucleic acids^18^ within a bilayered membrane, we postulate that these released cellular components are associated with and are likely contained within EVs.

The *S. aureus* cell envelope is comprised of a thick, highly cross-linked PGN layer, proteins, and glycopolymers like lipoteichoic acid, WTA, and CP. Highly cross-linked PGN serves as a barrier for EV biogenesis since treatment of *S. aureus* with a sublethal concentration of PenG or genetic inactivation of *pbp4* or *tagO* resulted in a significant increase in EV production and size. This inverse correlation between PGN cross-linking and EV yield was also observed with *S. aureus* strains MW2, COL, and Newman. WTA has been shown to be critical for PGN-cross-linking by regulating PBP4 localization to the septation site^28^. A secondary mechanism by which WTA regulates EV production is via its ability to control the activity of Atl and Sle1—not only by preventing their binding to *S. aureus* cell wall PGN^53,54^, but also by creating an acidic milieu that limits Atl PGN hydrolase activity^55^. Consequently, autolytic activity is not localized to the septum area in a *tagO* mutant but is spread throughout the cell surface, likely facilitating EV release. Schlag et al.^53^ reported that a *tagO* mutant showed an altered cell surface with bobble- and hairy-like protrusions, which may represent EVs. Although we do not yet fully understand the mechanism(s) of EV generation in Gram-positive bacteria, it seems logical that a poorly cross-linked cell wall or a cell wall lacking WTA would lessen the barrier to EV release and generate larger EVs as a result of larger pores within the PGN structure.

Autolysins that cleave the PGN barrier also impact the biogenesis of *S. aureus* EVs. Atl and Sle1 localize to the septum during cell division where they exhibit peptidoglycan hydrolase activity, resulting in separation of the daughter cells^53,54^. Sle1 is a 32 kDa protein comprised of an N terminal cell wall binding domain and a C terminal catalytic domain with N-acetyl muramyl-l-alanine amidase activity. In contrast, Atl is a 138 kDa bifunctional PGN hydrolase that is processed to yield a 62 kDa protein with amidase activity (similar to that of Sle1) and a 51 kDa protein with endo-β-N-acetyl glucosaminidase activity. Atl is also involved in cell wall turnover and penicillin- or detergent-induced bacterial autolysis. Deletion of *sle1*, but not *atl*, significantly reduced *S. aureus* EV production. Pasztor et al.^4^ reported that an SA113 *atl* mutant overexpressed eight putative secondary PGN hydrolases both at the transcriptional and at the protein levels, highlighting the supplementary role of these alternative autolysins in the absence of Atl. This observation may at least partially explain why JE2Δ*atl* and NewmanΔ*atl* showed only a modest reduction in EV yield. Mutation of *atl* only slightly reduced EV yield, but the average size of EVs from the mutant was smaller than that of WT JE2, suggesting that Atl is involved in EV biogenesis. Atl modulates the excretion of staphylococcal cytoplasmic proteins^4^, and it is likely that EV production at least partially explains the Atl-mediated shedding of cytoplasmic proteins in *S. aureus*.

Although both autolysin activities are localized to the *S. aureus* septum region, JE2 EVs are not confined to the septal region (Fig. 2f), and EVs have been visualized by others surrounding the bacterial surface^7,48,56^. A recent report demonstrated differential roles for Atl and Sle1 during cell division and separation^57^. Whereas Sle1 could be visualized over the entire septal surface, Atl localized only at the external (surface exposed) edge of the septum^58^. How autolysins modulate EV release from the cell wall or whether this process is spatially or temporally regulated remains to be determined.

We reported that *S. aureus* CP was shed from broth-grown *S. aureus* cells^59^, and it is feasible that EVs could serve as a vehicle to liberate CP from the cell envelope. The *Streptococcus pneumoniae* capsule was reported to hinder EV release in this pathogen^7^, whereas no effect was observed on EV yield in strains with or without the hyaluronic capsule of *Streptococcus pyogenes*^8^. Whether these streptococcal CPs are present as EV cargo in these pathogens was not addressed. Although EV yield varied among different isolates, we recovered similar quantities of EVs from isogenic *S. aureus* strains that varied only in CP production. The glucuronoxylomannan capsule of *Cryptococcus neoformans* has been identified as a component of EVs from this fungal pathogen^60^, and polysaccharide A from *Bacteroides fragilis* was shown to be packaged into OMVs that were capable of inducing immunomodulatory signaling in dendritic cells^46^. Ongoing studies in our laboratory will address whether *S. aureus* EV-host cell interactions impact the pathogenesis of staphylococcal disease.

We considered that *S. aureus* EVs could serve as a vaccine platform if their cytotoxicity was abrogated, and this was accomplished by purifying EVs from an ∆*agr*∆*spa* mutant of strain JE2. To enhance the protective efficacy of the ∆*agr*∆*spa* EV vaccine, non-toxic Hla_H35L_ and LukE were expressed in JE2∆*agr*∆*spa* under the control of the *agr-*derepressed *spa* promoter. Immunization with purified non-toxic Hla_H35L_ has been shown to prevent lethal pneumonia and lethal peritonitis and reduce the incidence of necrotic skin abscesses^61–63^. *S. aureus* leukocidins comprise a family of pore-forming cytolysins produced by *S. aureus* that target monocytes, lymphocytes, neutrophils, and macrophages—the very cells responsible for resolution of bacterial infection. These “eng-EVs” elicited antibodies in the sera of immunized mice that reacted with Hla and LukE by ELISA and neutralized the cytolytic activity of Hla, LukED, and HlgAB in vitro.

Immunization with eng-EVs provided significant protection against lethal sepsis provoked by USA300 strain LAC, a virulent PVL+ isolate. Because of a report that the presence of PVL modulates LukED-mediated *S. aureus* virulence in mice^41^, we challenged another group of immunized mice with USA500 strain NRS685, a PVL-negative MRSA bacteremia isolate. Immunization with eng-EVs, but not *∆agr∆spa* EVs, protected 50% of the mice against NRS685 lethal sepsis. Protective efficacy against additional *S. aureus* strains and in additional infection models remains to be evaluated. Overexpression of additional antigens that have been shown to protect mice against experimental *S. aureus* infections, such as MntC and FhuD2^35,36^, in second-generation eng-EVs may yield a more broadly protective vaccine. LC–MS/MS analysis of EVs from both WT JE2 and the ∆*agr*∆*spa* mutant strain contained multiple lipoproteins. As a predominant TLR2 ligand, lipoproteins have been increasingly used as adjuvant components^64,65^ because they are potent activators of host innate immunity and can mediate humoral and cell mediated immune responses. The self-adjuvanting composition of eng-EVs may provide it with a unique advantage over purified component vaccines.

**Fig. 7:**
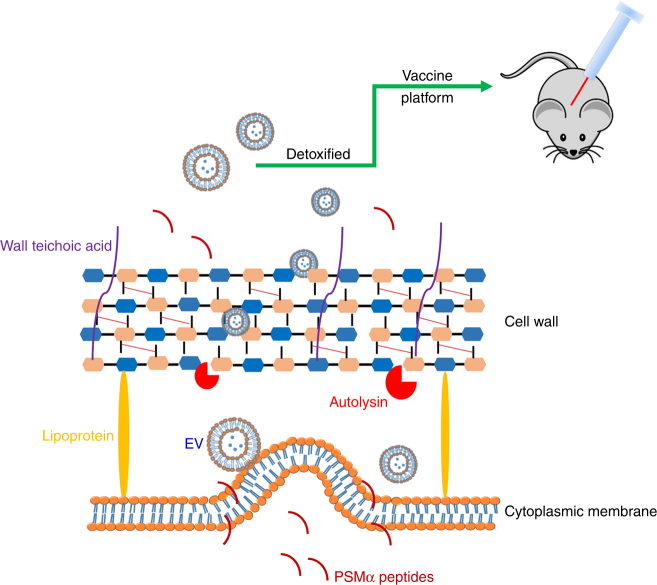
Proposed mechanisms underlying EV production by *Staphylococcus aureus*. EVs are generated from the cytoplasmic membrane, and this process is promoted by *S. aureus* PSMα peptides, which have surfactant-like activity, causing membrane disruption. Membrane-derived EVs must also traverse the highly cross-linked *S. aureus* peptidoglycan barrier, and the extent of cell wall cross-linking modulates the efficiency of EV production. Autolysins, such as Sle1, facilitate EV release by hydrolyzing peptidoglycan, particularly at sites of active cell division. We mutated *S. aureus* to render its EVs non-toxic, and then genetically engineered the mutants to package detoxified antigens in EVs. These recombinant EVs were immunogenic in mice and showed protective efficacy in a sepsis model of *S. aureus* infection

In summary, we have generated, purified, and characterized EVs isolated from *S. aureus* USA300, the predominant CA-MRSA clone in the United States. Our study revealed that *S. aureus* PSMs are central for EVs generation by targeting the cytoplasmic membrane. Likewise, the Sle1 autolysin was shown to be critical for the release of EVs from the *S. aureus* cell wall. Whereas mutations in Atl or CP production did not affect EV yield, PBP4 and WTA promoted PGN cross-linking and consequently diminished EV production. Our study elucidates certain mechanisms whereby *S. aureus* produces and sheds EVs (Fig. 7) and will ultimately further our understanding of bacterial physiology and pathogenesis. We designed and created eng-EVs as a vaccine platform against *S. aureus* infection. Detoxified EVs that over-produced Hla_H35L_ and LukE were immunogenic, elicited toxin neutralizing antibodies, and protected mice in a *S. aureus* lethal sepsis model, indicating that these naturally produced vesicles have potential as a noval vaccine platform.

## Methods

### Bacterial strains and plasmids

*S. aureus* isolates (listed in Supplementary Table 1) were cultivated overnight with aeration in tryptic soy broth (TSB; Difco) at 37 °C. *Escherichia coli* strain XL-10 (Agilent), used in DNA cloning experiments, was grown at 37 °C in Luria Broth (LB; Difco). *S. aureus—E. coli* shuttle vector pCU1^66^ was used for cloning and expression of appropriate genes in *S. aureus*, and pOS1-*hprK* was used for pSle1 complementation studies. Antibiotics were added in the following concentrations: PenG 0.2 μg ml^−1^, ampicillin (Amp; 100 μg ml^−1^), Em 5 μg ml^−1^, chloramphenicol (Cm; 10 μg ml^−1^), kanamycin (Kan; 50 μg ml^−1^), and tetracycline (Tet; 5 μg ml^−1^).

### DNA manipulation

Fey et al.^19^ derived *S. aureus* JE2 from the USA300 strain LAC by curing it of plasmids, rendering it sensitive to Em. The *agr* mutation (∆*agr*::*tetM*) was transduced from *S. aureus* RN6911^40^ to WT JE2 using bacteriophage ϕ80α with selection for Tet resistance. To construct the JE2 ∆*agr*∆*spa* double mutant, the *spa* mutation was transduced from JE2 (*spa*::*ermB*) to JE2*∆agr* by ϕ80α transduction. The *pbp4* mutation was transduced from JE2 (∆*pbp4*::*ermB*) to WT MW2 and COL by ϕ80α transduction with selection for Em resistance. All mutants were confirmed by PCR using the primers listed in Supplementary Table 2. ELISA results confirmed the phenotype of the *∆spa* mutant, and the *agr* mutant lost its hemolytic phenotype. To construct the WTA mutants, the *tagO* mutation was transduced from SA113∆*tagO* (pRB*tagO*) to WT JE2 and COL with ϕ80α with selection for Em resistance. Mutants were confirmed by PCR and acquisition of resistance to lysis by ϕ80α. To complement the JE2∆*sle1* mutant, a DNA fragment of 1005 bp containing the *sle1* gene was amplified from JE2 genomic DNA using the primer pair listed in Supplementary Table 2. The Sle1 expression plasmid was constructed by cloning the *sle1* gene under the control of the *hprK* promoter into the *E.coli—S. aureus* shuttle vector pOS1^67^. pSle1 was transformed into RN4220 by electroporation and then transduced to JE2∆*sle1* by ϕ80α transduction.

To construct a shuttle vector for expression of Hla_H35L_ and LukE, the *spa* promoter, *hla*_*H35L*_, and *lukE* genes were amplified from *S*. *aureus* strains JE2, DU1090 (pHla_H35L_), and FPR3757, respectively, using the primers listed in Supplementary Table 2. To drive the expression of *hla*_*H35L*_, its sequence was fused to the 3′ terminus of the *spa* promoter containing the ribosome binding site by overlapping PCR. The P_*spa*_-*hla*_*H35L*_ fusion sequence was cloned into the shuttle plasmid pCU1 with restriction enzymes HindIII and SalI. The amplified *lukE* sequence containing a ribosome binding site was inserted into pCU1 with restriction enzymes SalI and EcoRI. The resulting plasmid pCU1-P_*spa*_-*hla*_*H35L*_*-lukE* was verified by enzyme digestion and DNA sequencing. To construct JE2∆*spa*∆*agr* expressing non-toxic Hla_H35L_ and LukE, pCU1-P_*spa*_-*hla*_*H35L*_*-lukE* was transformed into RN4220 by electroporation and then transduced with ϕ80α to JE2∆*spa*∆*agr*, selecting for Cm resistance.

### Isolation and purification of EVs

*S. aureus* was cultivated in TSB with shaking to an OD_650 nm_ of 1.2. The culture supernatant was filtered and concentrated 25-fold with a 100-kDa tangential flow filtration system (Pall Corp.). The retentate was filtered again before centrifugation at 150,000×*g* for 3 h at 4 °C to pellet the vesicles and leave soluble proteins in the supernatant. The EV pellet was suspended in 40% Optiprep density gradient medium (Sigma) and overlaid with gradient layers of Optiprep ranging from 35 to 10%. After centrifugation at 139,000×*g* for 16 h at 4 °C, 1 ml fractions were removed sequentially from the top of the gradient. Each fraction was subjected to SDS-PAGE and stained with a Thermo Fisher silver staining kit. Fractions with a similar protein profile on SDS-PAGE were pooled, and the Optiprep medium was removed by diafiltration with PBS using an Amicon Ultra-50 Centrifugal Filter Unit. The diafiltered retentate was filtered (0.45 µm) and stored at 4 °C. EV protein concentrations were determined by using a Protein Assay Dye Reagent (Bio-Rad). EV samples were evaluated with a Nanobrook ZetaPALS potential analyzer (Brookhaven Instruments Corp.), and the data for size distribution and particle diameter were generated with ZetaPALS particle sizing software. Nanoparticle tracking analysis was performed by purifying EVs from 100 ml bacterial cultures, as described above. The number of EV particles recovered from individual cultures (and suspended in 1 ml PBS) was determined using a Nanosight NS300 Sub Micron Particle Imaging System (Malvern). A camera level of 12 and a gain of 1 was utilized to optimize data collection, and analyses were performed with Nanoparticle Tracking and Analysis software (NTA 3.1). Each sample was analyzed three times for 30 s at 20 °C using different fields of view. Frame sequences were analyzed under manual particle detection and tracking parameters (screen gain of 4 and detection threshold of 17).

### Transmission electron microscopy

For imaging *S. aureus* ultrathin sections, the cultures were fixed with 2.5% paraformaldehyde, 5% glutaraldehyde, and 0.06% picric acid in cacodylate buffer and postfixed with 1% osmium tetroxide/1.5% potassium ferrocyanide. After incubation in 1% uranyl acetate, samples were sequentially dehydrated in ethanol before they were soaked in propylene oxide and infiltrated overnight with propylene oxide and TAAB Epon. Ultrathin sections were stained with lead citrate. To image EVs, 5 µl *S. aureus* EVs were adsorbed for 1 min to a carbon coated grid that was made hydrophilic by a 30-s exposure to a glow discharge. The samples were stained with 0.75% uranyl formate. All samples were examined in a TecnaiG2 Spirit BioTWIN transmission electron microscope, and images were recorded with an AMT 2k CCD camera.

### Proteomic analysis of EVs by LC–MS/MS

*S. aureus* EVs (8–10 µg) were subjected to SDS-PAGE and stained with Coomassie Blue R-250. Gel sections were analyzed at the Taplin mass spectrometry facility at Harvard Medical School using an LTQ Orbitrap Velos Pro ion-trap mass spectrometer (Thermo Fisher). Peptide sequences (and hence protein identity) were determined by matching protein databases with the acquired fragmentation pattern using the software program Sequest (Thermo Fisher Scientific). Proteins were identified by a minimum of two peptides and at least one unique peptide. Sequence analysis was performed with a database containing protein sequences of the *S. aureus* USA300 FPR3757 genome downloaded from NCBIprot. The subcellular localization of each identified protein was predicted by PsortB v.3.0 (www.psort.org/psorb/).

### Real-time RT-PCR assay

*S. aureus* strains were cultivated in 5 ml TSB at 37 °C to an OD_650 nm_ of 0.9. After centrifugation at 4 °C, the bacterial cells were mixed with glass beads in 300 µl lysis buffer (RNeasy mini kit; Qiagen) and lysed by using a high speed Ultramat 2 Amalgamator (SDI, Inc.). Total RNA from the lysate supernatant was purified with the RNeasy mini kit (Qiagen), treated with DNase I (Invitrogen), and stored at −70 °C. cDNA was synthesized from 1 µg of bacterial RNA using a Protoscript II First Strand cDNA synthesis kit (New England Biolabs). A total of 50 ng of synthesized cDNA was subjected to Real-time RT-PCR using a Power Green PCR Master Mix (Applied Biosystems) with primers listed in Supplementary Table 2 and detected in a StepOnePlus Real-Time PCR System (Applied Biosystems). The relative transcriptional levels of *hla*_*H35L*_ and *lukE* were calculated using the ∆∆Ct method by normalizing to the 16S rRNA transcriptional level.

### Immunoblotting assays

For western blots, 10 µg *S. aureus* EVs were subjected to SDS-PAGE, transferred to nitrocellulose membranes, and blocked with PBS + 0.05% Tween-20 (PBST) and 1% skim milk for 1 h at room temperature (RT). After washing with PBST, the membranes were incubated with rabbit anti-LukS-PV (2 μg ml^−1^; IBT Bioservices; Cat. No. 04-0009) or mouse anti-Hla monoclonal antibody (mAb) 6C12 (1 μg ml^−1^; IBT Bioservices; Cat. No. 0210-005) overnight at 4 °C. The membranes were washed and incubated with HRP-conjugated goat anti-rabbit IgG (1:5000; ImmunoReagent, Inc.; Cat. No. GtxRb-004-DHRPX) or anti-mouse IgG (1:5000; ImmunoReagent, Inc.; Cat. No. GtxMu-004-DHRPX) for 2 h at RT before developing the blots using TMB membrane peroxidase substrate (Kirkegaard & Perry Laboratories, Inc). Purified Hla (List Biological Labs) and LukE (IBT Bioservices) were used as positive controls. Uncropped scans of the western blots are shown in Supplementary Fig. 6.

For EV dot blotting assays, intact or sonicated EVs were applied to nitrocellulose membranes using a 96-well dot blotter system (Bio-Rad). The membranes were immersed in PBST + 5% skim milk for 2 h before  blocking the staphylococcal IgG binding proteins (Spa and Sbi) by overnight incubation  at 4 °C with an irrelevant human IgG1 monoclonal antibody (10 µg ml^−1^) in PBST + 1% skim milk. The membrane was washed with PBST and incubated overnight at 4 °C with sera (diluted 1:1000 in PBST + 1% skim milk) pooled from mice immunized with EVs (see below) or murine mAbs^59^ to CP5 (4C2; 1.2 µg ml^−1^) or CP8 (5A6; 1.2 µg ml^−1^). After washes with PBST, the membrane was incubated with alkaline phosphatase (AP)-conjugated goat anti-mouse antibody (1:15,000; Sigma; Cat. No. A2429) at RT for 2 h. The membrane was washed with PBST and developed with AP membrane substrate (KPL).

### EV cytotoxicity

The relative toxicity of *S. aureus* EVs (1–20 µg ml^−1^) toward human A549 lung epithelial cells (ATCC), neutrophil-like HL60 cells (ATCC), and rabbit erythrocytes (Hardy Diagnostics) was assessed. A549 lung epithelial cells grown in a 96-well plate were incubated overnight at 37 °C with EVs or 1 µg ml^−1^ of purified Hla. Toxicity was assessed using an LDH cytotoxicity assay kit (Thermo Fisher Scientific). Differentiated HL60 cells (2 × 10^5^ cells) were seeded in a 96-well plate and treated with EVs or 1 µg ml^−1^ of PVL for 4 h at 37 °C. Cell viability was measured with a CellTiter kit (Promega). A 2% rabbit erythrocyte suspension was mixed with EVs or 1 µg ml^−1^ Hla in a 96-well plate for 1 h at 37 °C. The erythrocytes were pelleted by centrifugation, and hemolysis was recorded by measuring the OD_545 nm_ of the supernatant using an ELISA reader.

### Animal studies

Mouse experiments were carried out in accordance with the recommendations in the PHS Policy on Humane Care and Use of Laboratory Animals, and animal use protocols were approved by the Partners Healthcare Institutional Animal Care and Use Committee. Female Swiss Webster mice (4 weeks old; Charles River) were immunized by the subcutaneous route on days 0, 14, and 28 with 5 µg of ∆*agr* EVs, ∆*agr∆spa* EVs, or eng-EVs. Control animals were immunized similarly with BSA (Sigma). Blood was collected from the mice by tail vein puncture before each vaccination and again before challenge. Sera were diluted 1:100 and tested by ELISA on 96-well plates coated with 5 µg ml^−1^ sonicated WT EVs, 5 µg ml^−1^ LukE, or 1 µg ml^−1^ Hla. Immunized mice were inoculated with 0.8–2 × 10^8^ CFU *S. aureus* by intravenous (IV) tail vein injection 2 weeks after the third vaccination. Survival was monitored up to 15 days post challenge, and the data were analyzed using the log-rank test.

### Toxin neutralization assays

For the Hla neutralization assays, mouse serum samples were pre-incubated with native Hla for 1 h before the addition of 2% rabbit erythrocytes. After 1 h, the cytotoxicity of the serum-neutralized samples was measured by recording the OD_545 nm_ of the sample supernatants^68^. For leukocidin neutralization assays, blood was collected from healthy volunteers giving written informed consent, as approved by the Institutional Review Board of The Brigham and Women’s Hospital (Human Subject Assurance Number 00000484). Neutrophils were isolated from 10 ml blood using Polymorphprep (Accurate Chemical), washed, and suspended in RPMI (Invitrogen) containing 5% fetal bovine serum (Invitrogen). Sera from immunized mice were serially diluted and mixed with toxin concentrations yielding ~75% cell lysis (12.5 µg ml^−1^ LukED, 2.5 µg ml^−1^ PVL, 1 µg ml^−1^ HlgAB, or 2 µg ml^−1^ HlgCB (1∶1 S and F subunits). Samples were pre-incubated with leukocidins for 30 min at RT before the addition of neutrophils (1.2 × 10^5^ cells). After 2 h at 37 °C in 5% CO_2_, the cells were collected by centrifugation and suspended in fresh medium. Cell cytotoxicity was evaluated using a CellTiter kit (Promega). Percent neutralization was calculated using the formula: [% cytotoxicity of (toxin + cells)—% cytotoxicity of (serum + toxin + cells)].

### Data availability

Data supporting the findings of this manuscript are available from the corresponding author upon reasonable request. Mass spectrometry proteomics data were deposited in the ProteomeXchange Consortium (http://proteomecentral.proteomexchange.org) via the PRIDE partner repository with the data set identifier PXD007953.

## Electronic supplementary material

Supplementary Information

Description of Additional Supplementary Information

Supplementary Data 1

Supplementary Data 2
