# Supplementary material for: Release of Staphylococcus aureus extracellular vesicles and their application as a vaccine platform
Source: Nat Commun. 2018 Apr 11;9:1379. doi: 10.1038/s41467-018-03847-z (PMC5895597; doi:10.1038/s41467-018-03847-z)
Supplement: Supplementary file 1 — Supplementary Information [file 41467_2018_3847_MOESM1_ESM.docx]

**
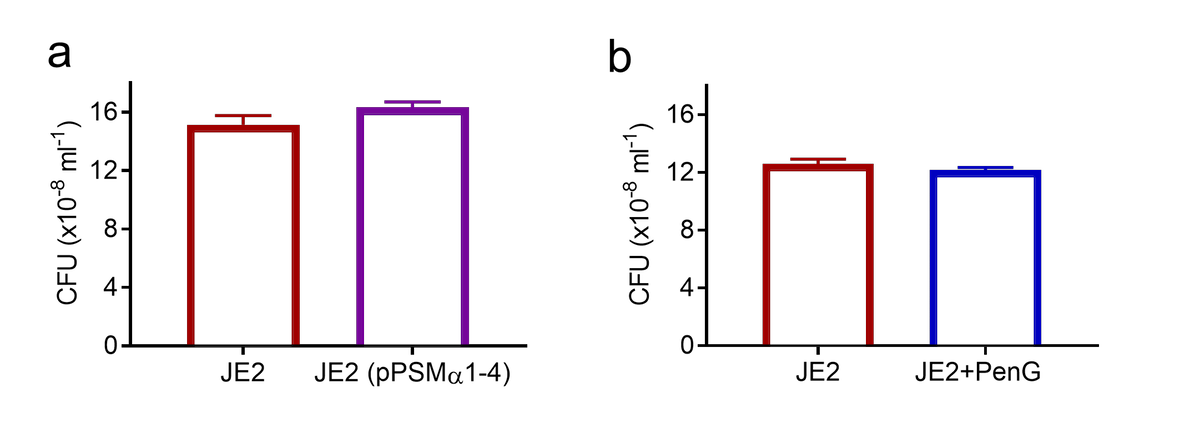
**

**Supplementary Figure 1** **Bacterial viability in cultures yielding significantly different extracellular vesicle amounts**

(**a**) *S. aureus* WT JE2 or JE2(pPSMα1-4) or (**b**) JE2 culture with or without added Penicillin G (0.2 μg ml^-1^) treatment were grown at 37°C until an OD_650nm_ of 1.2 was achieved. Bacterial cultures were serially diluted and plated in triplicate. Bacterial concentrations (CFU ml^-1^) were calculated from three independent experiments, and the data are expressed as mean ± s.e.m.

**
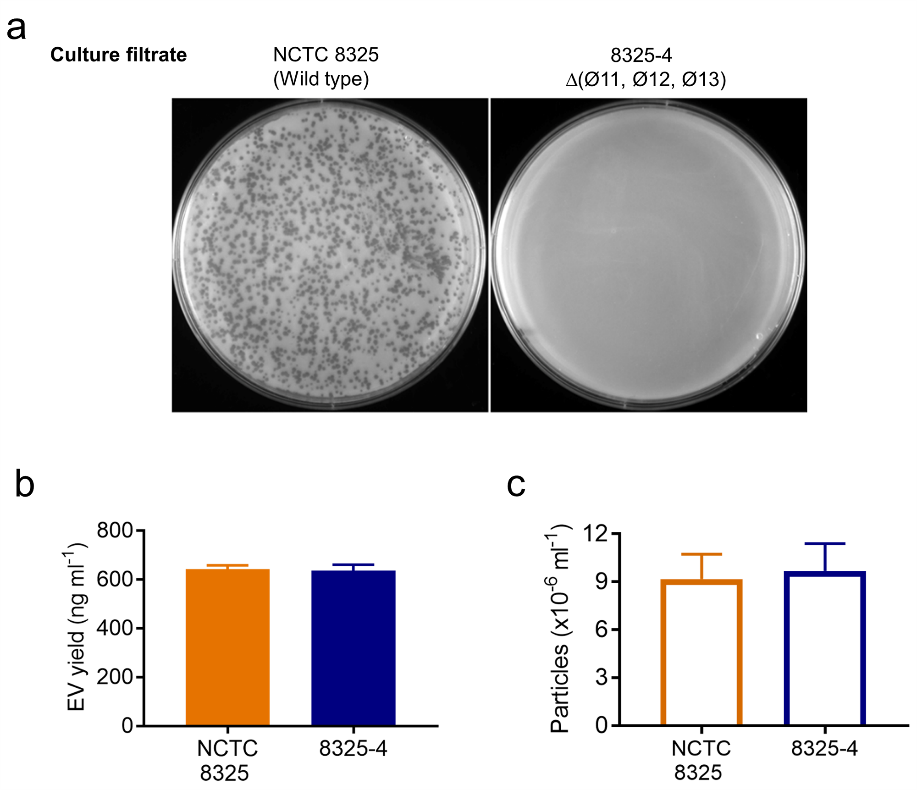
**

**Supplementary Figure 2** **Effect of *Staphylococcus aureus* prophages on extracellular vesicle production**

**(a)** NCTC 8325 and its prophage-free variant 8325 were grown in Luria-Bertani (LB) broth with 5 mM CaCl_2_ until an OD_650 nm_ of 1.6 was achieved. Bacterial cells were pelleted by centrifugation, and culture supernatants were passed through a 0.45 μm filter. 150 μl of each supernatant was mixed with the recipient strain RN4220 grown to early-log phase in LB medium with 5 mM CaCl_2_ and added to 4 ml soft agar (0.4% agar in LB medium) overlaid on an LB agar plate containing 5 mM CaCl_2_. **(b)** EV production from NCTC 8325 and its prophage-free strain 8325-4 was evaluated by quantification of total EV yield or **(c)** by EV quantification using nanoparticle tracking analysis. EV protein yield and EV particle quantification experiments were calculated from three independent experiments and expressed as mean ± s.e.m.

**
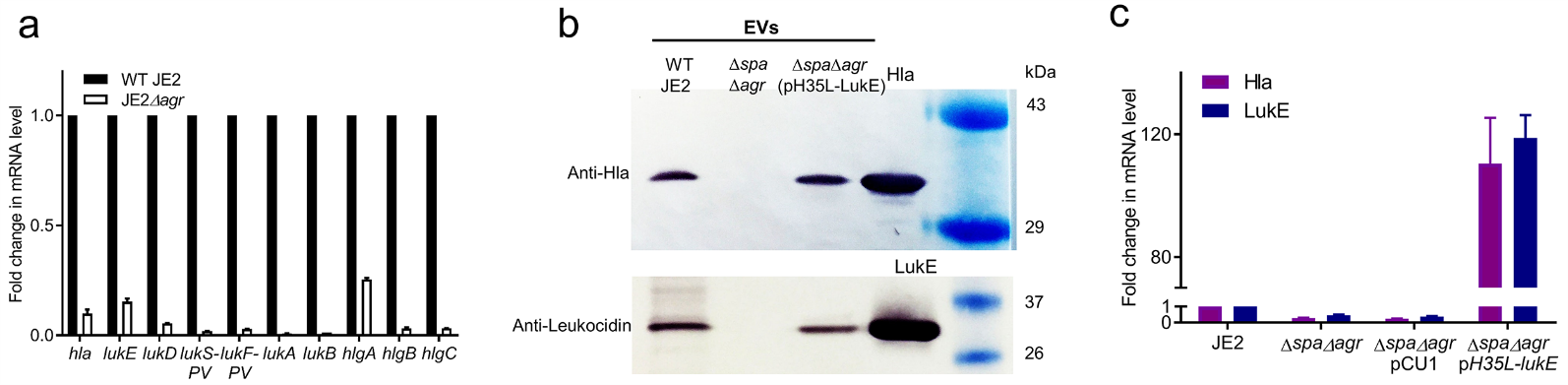
**

**Supplementary Figure 3** **Extracellular vesicles from strain JE2*∆agr∆spa* (pHla_H35L_-LukE) package recombinant Hla_H35L_ and LukE**

**(a)** Real time RT-PCR analysis revealed that the mRNA levels of *hla* and genes encoding the leukocidin subunits were dramatically reduced in an *agr* mutant compared to the WT strain JE2. Data are expressed as mean ± s.e.m. relative to the WT strain, and each strain was tested in three replicates. **(b)** Purified EV samples were subjected to SDS-PAGE. Western blot analysis revealed that Hla_H35L_ and LukE were detected in EVs from recombinant strain JE2*∆agr∆spa* (pHla_H35L_-LukE) but not in EVs prepared from JE2∆*agr*∆*spa*. **(c)** Real time RT-PCR analysis revealed that the expression of *hla_H35L_* and *lukE* was enhanced ~100-fold in JE2∆*agr*∆*spa* (pH35L-LukE) compared to the parental strain JE2. Data are expressed as mean ± s.e.m., and each group was tested in three replicates.

**
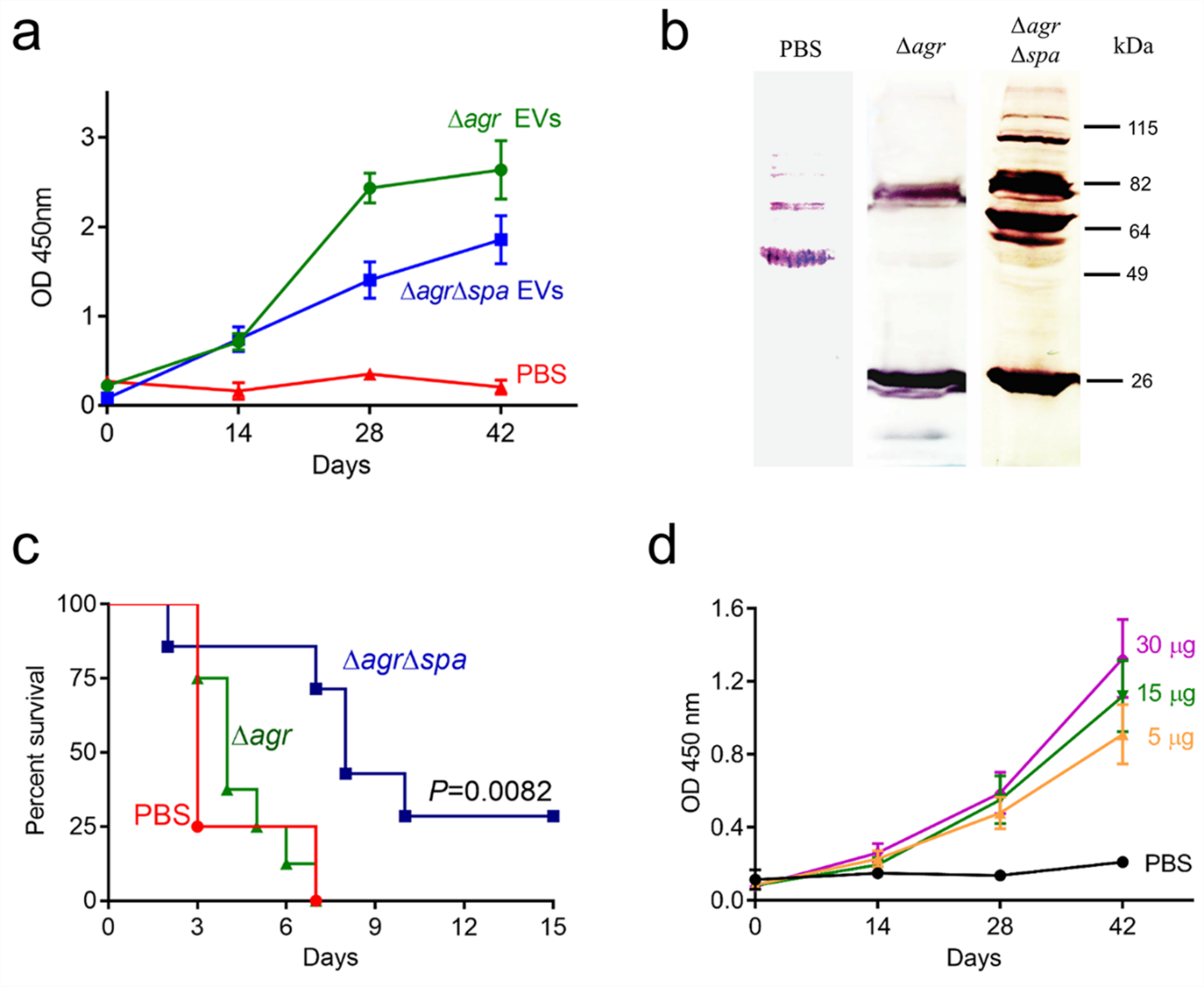
**

**Supplementary Figure 4** **Evaluation of the protective efficacy of *Staphylococcus aureus* extracellular vesicles**

**(a**) Antibody levels in sera (diluted 1:100) from mice immunized with different EVs were analyzed on ELISA plates coated with sonicated JE2 EVs. Data are expressed as mean ± s.e.m., and each serum sample was tested in duplicate. **(b**) USA300 strain FPR3757 cell lysates were subjected to SDS-PAGE. Western blot analysis was performed with sera from mice immunized with different EV preparations. **(c**) EV-immunized mice (n=8) were challenged IV with 2x10^8^ CFU strain FPR3757. Mice immunized with EVs were compared to mice given PBS, and survival was analyzed with the log rank test. **(d**) Antibody levels in sera (diluted 1:100) from mice immunized with different doses of JE2*∆agr∆spa* EVs with alum were analyzed on ELISA plates coated with sonicated JE2 EVs. Data are expressed as mean ± s.e.m., and each serum sample was tested in duplicate.


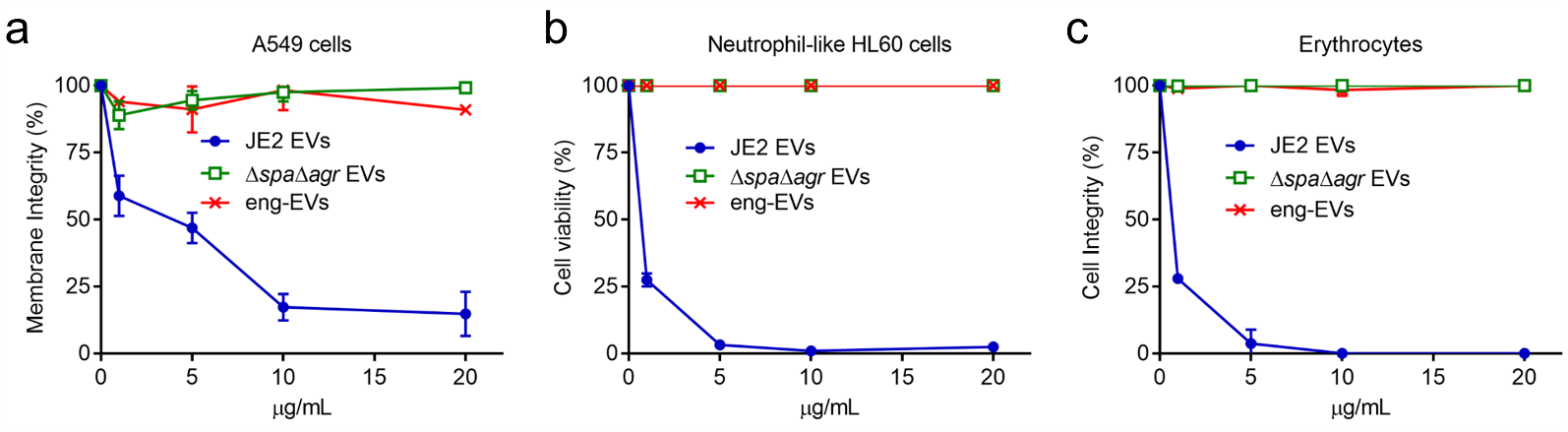


**Supplementary Figure 5** **Cytotoxicity of *Staphylococcus aureus* extracellular vesicles**

**(a**) Human lung A549 lung epithelial cells, **(b)** neutrophil-like HL60 cells, and **c)** rabbit erythrocytes were treated with different concentration of EVs produced by WT JE2, JE2*∆agr∆spa*, and eng-EVs, and cell cytotoxicity was evaluated. Each sample was tested in duplicate, and two independent experiments were performed with similar results. A representative experiment is shown.


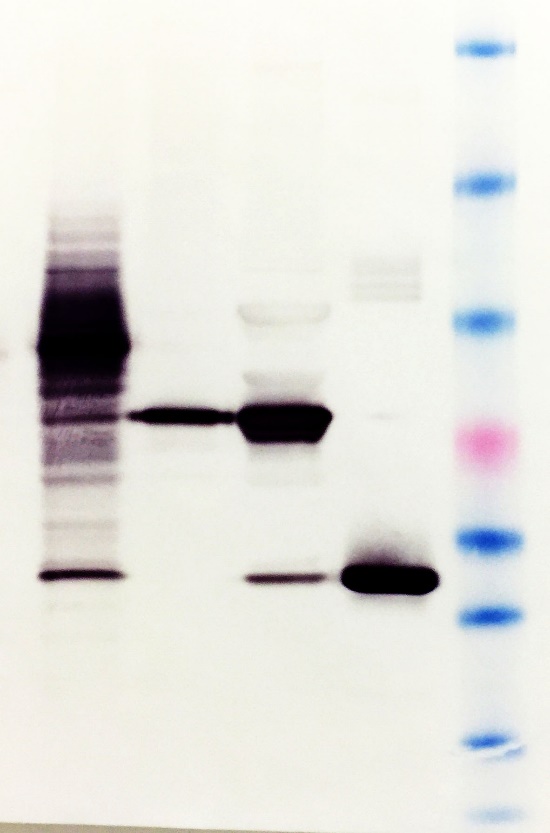


b


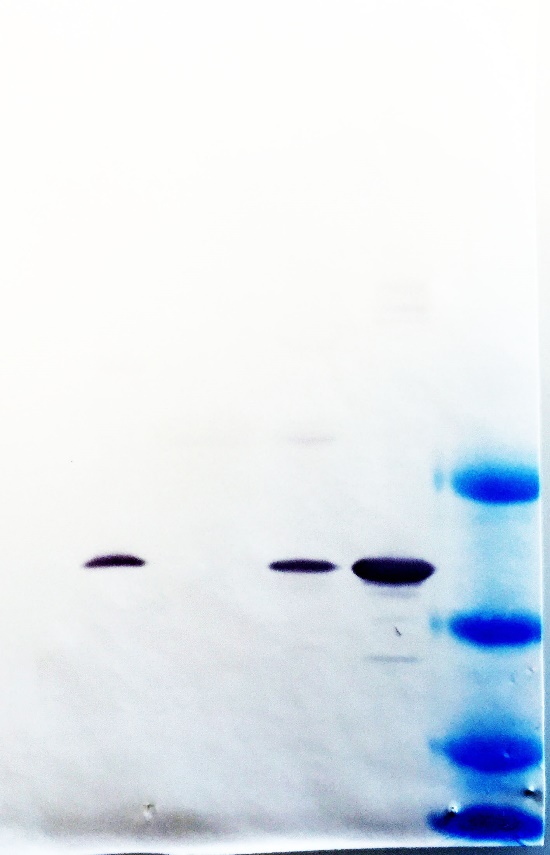


a

**Supplementary Figure 6 The full immunoblot data for Supplementary Figure 3b**

**(a)** Western blot analysis for Hla from different EV samples; **(b)** Western blot analysis for LukE from different EV samples

**Supplementary Table 1. Strains used in this study**

| *S. aureus* strain | Description^a^ | Reference | |
| --- | --- | --- | --- |
| JE2 | USA300 LAC cured of 3 plasmids | | [^1^](#_ENREF_1) |
| JE2 ∆*pbp4* | JE2 *pbp4*::*bursa aurealis*, Em^r^ | | [^1^](#_ENREF_1) |
| JE2 ∆*tagO* | JE2 *tagO*::ErmB, Em^r^ | | This study |
| JE2 ∆*atl* | JE2 *atl*::*bursa aurealis*, Em^r^ | | [^1^](#_ENREF_1) |
| JE2 ∆*sle1* | JE2 *sle1*::*bursa aurealis*, Em^r^ | | [^1^](#_ENREF_1) |
| JE2 ∆*sle1 (*pSle1*)* | JE2 *∆sle1* (pOS1*-hprK-sle1*) | | This study |
| JE2 ∆*sle1*(pOS-hprK)) | JE2 *∆sle1* (carrying empty vector) | | This study |
| JE2(pPSMα1-4) | JE2 (pTX_∆_*psmα1-4*) | | This study |
| JE2 ∆*agr* | JE2 *agr*::tetM, Tc^r^ | | This study |
| JE2 ∆*spa* | JE2 *spa*::*bursa aurealis*, Em^r^ | | [^1^](#_ENREF_1) |
| JE2 ∆*agr*∆*spa* | JE2 *agr*::tetM *spa*::*bursa aurealis* , Em^r^, Tc^r^ | | This study |
| JE2 ∆*agr*∆*spa* (pCU1-Hla_H35L_/LukE) | Expression of Hla_H35L_ and LukE | | This study |
| COL | MRSA strain | [^2^](#_ENREF_2) | |
| COL ∆*pbp4* | COL *pbp4*::*bursa aurealis*, Em^r^ | This study | |
| COL ∆*tagO* | COL *tagO*::ErmB, Em^r^ | This study | |
| MW2 | USA400 MRSA strain | [^3^](#_ENREF_3) | |
| MW2 ∆*pbp4* | MW2 *pbp4*::*bursa aurealis*, Em^r^ | This study | |
| Newman | capsule type 5 | [^4^](#_ENREF_4) | |
| Newman ∆*tagO* | Newman *tagO*::ErmB, Em^r^ | [^5^](#_ENREF_5) | |
| Newman ∆*dltA* | Newman *dltA*::spc, Spc^r^ | [^5^](#_ENREF_5) | |
| Newman ∆*atl* | Newman *atl*::ErmB, Em^r^ | [^4^](#_ENREF_4) | |
| Newman ∆*cap5O* | Lacks type 5 capsule production | [^6^](#_ENREF_6) | |
| 6850 | capsule type 8 | [^7^](#_ENREF_7) | |
| 6850 ∆*capHIJK* | 6850 *capHIJK*::ErmB, Em^r^ | This study | |
| 923 | USA300 strain, CP5- | [^8^](#_ENREF_8) | |
| 923/pCap17 | USA300 strain, CP5+ | [^8^](#_ENREF_8) | |
| SA113 ∆*tagO* (pRBtagO) | Complemented ∆*tagO;* WTA positive | [^5^](#_ENREF_5) | |
| LAC | USA300 | [^9^](#_ENREF_9) | |
| LAC ∆*psmα* | LAC *psmα1-4*::spc, Spc^r^ | [^9^](#_ENREF_9) | |
| LAC ∆*psmβ* | LAC *psmβ1-2*::spc, Spc^r^ | [^9^](#_ENREF_9) | |
| LAC ∆*psmα∆psmβ* | *psmα* and *psmβ* double deletions in LAC, Spc^r^ | [^9^](#_ENREF_9) | |
| LAC ∆*psmα* (pPSMα1-4) | LAC ∆*psmα* (pTX_∆_*psmα1-4*) | [^9^](#_ENREF_9) | |
| LAC ∆*psmα* (pTX*_∆_*) | pTX_∆_ empty plasmid in LAC ∆*psmα* | [^9^](#_ENREF_9) | |
| MN8 | ST30, capsule type 8 | [^10^](#_ENREF_10) | |
| FPR3757 | USA300 MRSA strain | [^11^](#_ENREF_11) | |
| NCTC 8325 | WT strain lysogenized with 11, 12, and 13 | [^12^](#_ENREF_12) | |
| 8325-4 | phage-cured derivate of NCTC 8325 | [^12^](#_ENREF_12) | |
| RN4220 | Restriction-deficient mutant of 8325-4 | [^13^](#_ENREF_13) | |
| RN6911 | *agr* mutant of RN6390B, Tc^r^ | [^14^](#_ENREF_14) | |
| DU1090 | 8325-4 expressing nontoxic Hla_H35L_ | [^15^](#_ENREF_15) | |
| NRS685 | USA500 MRSA strain | [^16^](#_ENREF_16) | |

^a^ Em, erythromycin; Spc, spectinomycin; Tc, tetracycline

**Supplementary Table 2 Primers used for the study**

| Primer | Sequence^a^ |
| --- | --- |
| *spa*-fwd | ATTGCGTTGTTCTTCGTT |
| *spa*-rev | CTAGGTGTAGGTATTGCATC |
| *agr*-fwd | TCGCCCTTTGCAAATGAATG |
| *agr*-rev | TTGCGCCATAGGATTGTAGA |
| *pbp4*-fwd | ACCTCTTCTGTTTGAAATTTATAGT |
| *pbp4*-rev | GTCCGTTTTTAGTATGTTTTATTTTCTT |
| *tagO*-fwd | TCGATGAAGGTGAATAAATGG |
| *tagO*-rev | CCAAAGCAGTTACCTTTCG |
| *Atl-*fwd | AAGCAGCTGAGACGACACAA |
| *Atl-*rev | TTGCTGTTTTTGGTTGGACA |
| *sle1-*fwd | AGAATGTTAGGAAAGTTAAGCAAGA |
| *sle1-*rev | TTTATATACGTAAGACTTTAGTGA |
| *sle1* for expression*-*fwd | GCG**CATATG**CAAAAAAAAGTAATTG |
| *sle1* for expression*-*rev | GCG**CTCGAG**TTAGTGAATATATCTA |
| *cap8-fwd* | TGAGGATAGCGATTCTTGGCGCT |
| *cap8-rev* | TGC GTC AAC CCA GCT GTG TCC T |
| 16s rRNA-fwd | TAACGGCTTACCAAGGCAAC |
| 16s rRNA-rev | CGGAAGATTCCCTACTGCTG |
| Hla-fwd | AGCGAAGAAGGTGCTAACAAAAGT |
| *hla*-rev | GTTGCAACTGTACCTTAAAGGCT |
| *LukE*-fwd | GGACCATTTGGACTTTGTACGAA |
| *LukE*-rev | AATTTGTTACGCCTGATGGAAAA |
| *LukE* for expression*-*fwd | GCA**GTCGAC**AGGGAGGTTTTAAACATGTTTAAGAAAAAAATGTTAGCTGC |
| *LukE* for expression*-*rev | GCG**GAATTC**TTAATTATGTCCTTTCACTTTAATTTCGTG |
| P*spa-H35L*-fwd | CGC**AAGCTT**ACGCAAGTGTGCTGTATTCTAAAG |
| P*spa*-*H35L*-R1 | TATACGTGTTTTCATATTAATACCCCCTGT |
| P*spa*-*H35L*-F1 | GGTATTAATATGAAAACACGTATAGTCAGCTC |
| P*spa-H35L*-rev | CGG**GTCGAC**TTAATTTGTCATTTCTTCTTTTTCCCAA |

^a^Restriction enzyme sites are highlighted in bold.

**References**

1. Fey, P. D. *et al.* A genetic resource for rapid and comprehensive phenotype screening of nonessential *Staphylococcus aureus* genes. *MBio* **4**, e00537-00512 (2013).

2. De Lencastre, H. *et al.* Antibiotic resistance as a stress response: complete sequencing of a large number of chromosomal loci in *Staphylococcus aureus* strain COL that impact on the expression of resistance to methicillin. *Microb Drug Resist* **5**, 163-175 (1999).

3. Baba, T. *et al.* Genome and virulence determinants of high virulence community-acquired MRSA. *Lancet* **359**, 1819-1827 (2002).

4. Misawa, Y. *et al.* *Staphylococcus aureus* colonization of the mouse gastrointestinal tract Is modulated by wall teichoic acid, capsule, and surface proteins. *PLoS Pathog* **11**, e1005061 (2015).

5. Weidenmaier, C., McLoughlin, R. M. & Lee, J. C. The zwitterionic cell wall teichoic acid of *Staphylococcus aureus* provokes skin abscesses in mice by a novel CD4+ T-cell-dependent mechanism. *PLoS One* **5**, e13227 (2010).

6. Pohlmann-Dietze, P. *et al.* Adherence of *Staphylococcus aureus* to endothelial cells: influence of capsular polysaccharide, global regulator *agr*, and bacterial growth phase. *Infect Immun* **68**, 4865-4871 (2000).

7. Fraunholz, M. *et al.* Complete genome sequence of *Staphylococcus aureus* 6850, a highly cytotoxic and clinically virulent methicillin-sensitive strain with distant relatedness to prototype strains. *Genome Announc* **1**, e00775-00713 (2013).

8. Boyle-Vavra, S. *et al.* USA300 and USA500 clonal lineages of *Staphylococcus aureus* do not produce a capsular polysaccharide due to conserved mutations in the *cap5* locus. *MBio* **6**, e02585-02514 (2015).

9. Wang, R. *et al.* Identification of novel cytolytic peptides as key virulence determinants for community-associated MRSA. *Nat Med* **13**, 1510-1514 (2007).

10. Liu, B., Park, S., Thompson, C. D., Li, X. & Lee, J. C. Antibodies to *Staphylococcus aureus* capsular polysaccharides 5 and 8 perform similarly in vitro but are functionally distinct in vivo. *Virulence* **8**, 859-874 (2017).

11. Diep, B. A. *et al.* Complete genome sequence of USA300, an epidemic clone of community-acquired meticillin-resistant *Staphylococcus aureus*. *Lancet* **367**, 731-739 (2006).

12. Novick, R. Properties of a cryptic high-frequency transducing phage in *Staphylococcus aureus*. *Virology* **33**, 155-166 (1967).

13. Nair, D. *et al.* Whole-genome sequencing of *Staphylococcus aureus* strain RN4220, a key laboratory strain used in virulence research, identifies mutations that affect not only virulence factors but also the fitness of the strain. *J Bacteriol* **193**, 2332-2335 (2011).

14. Novick, R. P. *et al.* Synthesis of staphylococcal virulence factors is controlled by a regulatory RNA molecule. *EMBO J* **12**, 3967-3975 (1993).

15. Pozzi, C. *et al.* Opsonic and protective properties of antibodies raised to conjugate vaccines targeting six *Staphylococcus aureus* antigens. *PLoS One* **7**, e46648 (2012).

16. Benson, M. A. *et al.* Evolution of hypervirulence by a MRSA clone through acquisition of a transposable element. *Mol Microbiol* **93**, 664-681 (2014).
