# Supplementary material for: Release of Staphylococcus aureus extracellular vesicles and their application as a vaccine platform
Source: Nat Commun. 2018 Apr 11;9:1379. doi: 10.1038/s41467-018-03847-z (PMC5895597; doi:10.1038/s41467-018-03847-z)
Supplement: Supplementary file 2 — Description of Additional Supplementary Information [file 41467_2018_3847_MOESM2_ESM.docx]

**Description of Additional Supplementary Files**

File Name: Supplementary Data 1

Description: Proteins identified by LC/MS-MS in EVs purified from WT JE2

File Name: Supplementary Data 2

Description: Proteins that were identified by LC-MS/MS in EVs purified from JE2∆agr∆spa mutant
