# Supplementary material for: Release of Staphylococcus aureus extracellular vesicles and their application as a vaccine platform
Source: Nat Commun. 2018 Apr 11;9:1379. doi: 10.1038/s41467-018-03847-z (PMC5895597; doi:10.1038/s41467-018-03847-z)
Supplement: Supplementary file 3 — Supplementary Data 1 [file 41467_2018_3847_MOESM3_ESM.docx]

**Release of *Staphylococcus aureus* extracellular vesicles and their application as a vaccine platform**

Wang et al.

**Supplementary Data 1 Proteins identified by LC/MS-MS in EVs purified from WT JE2**

| Protein^a^ | | | | | | Gene name | | | MW  (kDa) | | | | | | Predicted localization^b^ | | Unique peptides^c^ | | | Coverage (%)^d^ | |  |  |  |  |  |
| --- | --- | --- | --- | --- | --- | --- | --- | --- | --- | --- | --- | --- | --- | --- | --- | --- | --- | --- | --- | --- | --- | --- | --- | --- | --- | --- |
| DNA-directed RNA polymerase, beta subunit | | | | | | *rpoC* | | | 135.2 | | | | | | C | | 49 | | | 49.21 | | | | |  |  |
| DNA-directed RNA polymerase, beta subunit | | | | | | *rpoB* | | | 133.14 | | | | | | C | | 36 | | | 39.9 | | | | |  |  |
| dihydrolipoamide acetyltransferase | | | | | | *pdhC* | | | 46.35 | | | | | | C | | 31 | | | 74.88 | | | | |  |  |
| 2-oxoisovalerate dehydrogenase, E3 component, lipoamide dehydrogenase | | | | | | *lpdA* | | | 49.41 | | | | | | C | | 23 | | | 59.83 | | | | |  |  |
| pyruvate dehydrogenase E1 component, alpha subunit | | | | | | *pdhA* | | | 41.36 | | | | | | C | | 26 | | | 77.3 | | | | |  |  |
| polyribonucleotide nucleotidyltransferase | | | | | | *pnp* | | | 77.31 | | | | | | C | | 25 | | | 45.27 | | | | |  |  |
| phosphopyruvate hydratase | | | | | | *eno* | | | 47.09 | | | | | | C | | 24 | | | 70.51 | | | | |  |  |
| pyruvate dehydrogenase E1 component, beta subunit | | | | | | *pdhB* | | | 35.22 | | | | | | C | | 24 | | | 74.77 | | | | |  |  |
| pyruvate carboxylase | *pyc* | | | | | | | | | 128.44 | | | | | | C | 18 | | | 19.65 | | | | | |  |
| Putative formate dehydrogenase | SAUSA300_2258 | | | | | | | | | 111.17 | | | | | | C | 13 | | | 19.11 | | | | | |  |
| glucose-6-phosphate isomerase | | | | | | *pgi* | | | 49.78 | | | | | | C | | 28 | | | 67.95 | | | | |  |  |
| Catalase | | | | | | *katA* | | | 58.34 | | | | | | C | | 19 | | | 49.5 | |  |  |  |  |  |
| phenylalanyl-tRNA synthetase, beta subunit | | | | | | *pheT* | | | 88.92 | | | | | | C | | 18 | | | 31 | |  |  |  |  |  |
| glutamine synthetase, type I | | | | | | *glnA* | | | 50.81 | | | | | | C | | 15 | | | 43.95 | |  |  |  |  |  |
| cytosol aminopeptidase | | | | | | *ampA* | | | 54.09 | | | | | | C | | 14 | | | 38.09 | |  |  |  |  |  |
| glutamyl-aminopeptidase | | | | | | SAUSA300_2400 | | | 39.17 | | | | | | C | | 13 | | | 64.8 | |  |  |  |  |  |
| glyceraldehyde-3-phosphate dehydrogenase, type I | | | | | | *gap* | | | 36.26 | | | | | | C | | 13 | | | 52.38 | |  |  |  |  |  |
| delta-aminolevulinic acid dehydratase | | | | | | *hemB* | | | 36.56 | | | | | | C | | 12 | | | 49.07 | |  |  |  |  |  |
| ferritin-like protein | | | | | | *ftnA* | | | 19.58 | | | | | | C | | 10 | | | 40.96 | |  |  |  |  |  |
| oligoendopeptidase F | | | | | | *pepF* | | | 69.78 | | | | | | C | | 10 | | | 22.16 | |  |  |  |  |  |
| translation elongation factor G | | | | | | *fusA* | | | 76.56 | | | | | | C | | 10 | | | 18.18 | |  |  |  |  |  |
| Oligoendopeptidase F | | | | | | SAUSA300_0902 | | | 69.78 | | | | | | C | | 10 | | | 22.26 | |  |  |  |  |  |
| L-lactate dehydrogenase 2 | | | | | | *ldh2* | | | 34.4 | | | | | | C | | 9 | | | 38.56 | |  |  |  |  |  |
| ATP synthase F1, alpha subunit | | | | | | *atpA* | | | 54.55 | | | | | | C | | 9 | | | 22.91 | |  |  |  |  |  |
| Ribonuclease J2 | | | | | | *rnj2* | | | 62.56 | | | | | | C | | 9 | | | 19.21 | |  |  |  |  |  |
| translation elongation factor Tu | | | | | | *tuf* | | | 43.08 | | | | | | C | | 8 | | | 30.2 | |  |  |  |  |  |
| putative glutamyl aminopeptidase | | | | | | SAUSA300_1261 | | | 37.83 | | | | | | C | | 9 | | | 30.9 | |  |  |  |  |  |
| glutamyl-aminopeptidase | | | | | | SAUSA300_1691 | | | 39.76 | | | | | | C | | 8 | | | 34.08 | |  |  |  |  |  |
| L-lactate dehydrogenase | | | | | | *ldh1* | | | 34.56 | | | | | | C | | 8 | | | 33.75 | |  |  |  |  |  |
| phenylalanyl-tRNA synthetase, alpha subunit | | | | | | *pheS* | | | 40.08 | | | | | | C | | 8 | | | 26.99 | |  |  |  |  |  |
| Ribonuclease J 1 | | | | | | *rnj1* | | | 62.63 | | | | | | C | | 8 | | | 20.53 | |  |  |  |  |  |
| methionine--tRNA ligase | | | | | | *metG* | | | 74.83 | | | | | | C | | 8 | | | 15.22 | |  |  |  |  |  |
| inosine-5'-monophosphate dehydrogenase | | | | | | *guaB* | | | 52.82 | | | | | | C | | 7 | | | 21.72 | |  |  |  |  |  |
| formate acetyltransferase | | | | | | *pflB* | | | 84.81 | | | | | | C | | 7 | | | 14.69 | |  |  |  |  |  |
| D-alanine aminotransferase | | | | | *dat* | | | | | | 31.87 | | C | | | | | | 7 | 30.5 |  |  |  |  |  |  |
| 6-phosphogluconate dehydrogenase, decarboxylating | | | | | *gnd* | | | | | | 51.77 | | C | | | | | | 7 | 23.29 |  |  |  |  |  |  |
| 50S ribosomal protein L19 | | | | | | *rplS* | | | 13.35 | | | | | | C | | 6 | | | 39.66 | |  |  |  |  |  |
| 50S ribosomal protein L2 | | | | | | *rplB* | | | 30.14 | | | | | | C | | 6 | | | 33.57 | |  |  |  |  |  |
| chorismate, mutase/phospho-2-dehydro-3-deoxyheptonate aldolase | | | | | | SAUSA300_1683 | | | 40.59 | | | | | | C | | 6 | | | 22.31 | |  |  |  |  |  |
| ATP-dependent Clp protease, proteolytic subunit clpP | | | | | | *clpP* | | | 21.5 | | | | | | C | | 5 | | | 34.87 | |  |  |  |  |  |
| purine nucleoside phosphorylase | | | | | | *deoD* | | | 25.89 | | | | | | C | | 5 | | | 34.32 | |  |  |  |  |  |
| riboflavin synthase, beta subunit | | | | | | *ribH* | | | 16.39 | | | | | | C | | 5 | | | 50.65 | |  |  |  |  |  |
| aldehyde dehydrogenase | | | | | | *aldA2* | | | 51.71 | | | | | | C | | 5 | | | 15.25 | |  |  |  |  |  |
| glycosyl transferase, group 1 family protein | | | | | | SAUSA300_0550 | | | 58.38 | | | | | | C | | 5 | | | 12.9 | |  |  |  |  |  |
| glycosyl transferase, group 1 family protein | | | | | | SAUSA300_0549 | | | 57.16 | | | | | | C | | 5 | | | 10.82 | |  |  |  |  |  |
| general stress protein 20U | | | | | | *dps* | | | 16.68 | | | | | | C | | 4 | | | 42.86 | |  |  |  |  |  |
| 50S ribosomal protein L17 | | | | | | *rplQ* | | | 13.74 | | | | | | C | | 4 | | | 40.16 | |  |  |  |  |  |
| isoleucyl-tRNA synthetase | | | | | | *ileS* | | | 104.82 | | | | | | C | | 4 | | | 4.36 | |  |  |  |  |  |
| 2-oxoglutarate dehydrogenase, E2 component, dihydrolipoamide succinyltransferase | | | | | | *sucB* | | | 46.64 | | | | | | C | | 4 | | | 9.48 | |  |  |  |  |  |
| 30S ribosomal protein S2 | | | | | *rpsB* | | | 29.08 | | | | | C | | | | | | 4 | 18.04 |  |  |  |  |  |  |
| glutamyl-tRNA synthetase | | | | | *gltX* | | | 56.25 | | | | | C | | | | | | 4 | 11.16 |  |  |  |  |  |  |
| tryptophanyl-tRNA synthetase | | | | | | *trpS* | | | 36.89 | | | | | | C | | 4 | | | 16.72 | |  |  |  |  |  |
| phosphate acetyltransferase | | | | | | *pta* | | | 34.93 | | | | | | C | | 4 | | | 22.56 | |  |  |  |  |  |
| 2-oxoglutarate dehydrogenase E1 component | | | | | | *odhA* | | | 105.28 | | | | | | C | | 3 | | | 6.44 | |  |  |  |  |  |
| fructose bisphosphate aldolase | | | | | | *fba* | | | 30.82 | | | | | | C | | 3 | | | 16.08 | |  |  |  |  |  |
| phosphoglycerate kinase | | | | | | *pgk* | | | 42.58 | | | | | | C | | 3 | | | 10.61 | |  |  |  |  |  |
| pyruvate kinase | | | | | | *pyk* | | | 63.06 | | | | | | C | | 3 | | | 7.52 | |  |  |  |  |  |
| exodeoxyribonuclease VII, large subunit | | | | | | *xseA* | | | 50.86 | | | | | | C | | 3 | | | 5.39 | |  |  |  |  |  |
| alcohol dehydrogenase | | | | | | *adh* | | | 36.02 | | | | | | C | | 3 | | | 12.5 | |  |  |  |  |  |
| glucose-6-phosphate 1-dehydrogenase | | | | | | *zwf* | | | 56.93 | | | | | | C | | 3 | | | 7.89 | |  |  |  |  |  |
| S1 RNA binding domain protein | | | | | | SAUSA300_2021 | | | 80.88 | | | | | | C | | 3 | | | 5.03 | |  |  |  |  |  |
| 2-oxoisovalerate dehydrogenase, E2 component, dihydrolipoamide acetyltransferase | | | | | | SAUSA300_1464 | | | 46.71 | | | | | | C | | 3 | | | 8.02 | |  |  |  |  |  |
| ABC transporter substrate-binding protein | | | | | | SAUSA300_0598 | | | 31.07 | | | | | | C | | 3 | | | 15.27 | |  |  |  |  |  |
| DNA-directed RNA polymerase alpha subunit | | | | | | *rpoA* | | | 34.99 | | | | | | C | | 3 | | | 15.92 | |  |  |  |  |  |
| CTP synthase | | | | | | *pyrG* | | | 59.95 | | | | | | C | | 2 | | | 3.73 | |  |  |  |  |  |
| putative fructose-1,6-bisphosphatase | | | | | | *fbp* | | | 76.13 | | | | | | C | | 2 | | | 3.67 | |  |  |  |  |  |
| 30S ribosomal protein S4 | | | | | | *rpsD* | | | 23 | | | | | | C | | 2 | | | 8.5 | |  |  |  |  |  |
| endonuclease IV | | | | | | *nfo* | | | 33.14 | | | | | | C | | 2 | | | 8.45 | |  |  |  |  |  |
| triosephosphate isomerase | | | | | | *tpiA* | | | 27.24 | | | | | | C | | 2 | | | 9.88 | |  |  |  |  |  |
| staphylococcal accessory regulator A | | | | | | *sarA* | | | 14.71 | | | | | | C | | 2 | | | 16.94 | |  |  |  |  |  |
| ribosomal protein S12 | | | | | | *rpsL* | | | 15.28 | | | | | | C | | 2 | | | 14.6 | |  |  |  |  |  |
| ribosomal protein L1 | | | | | | *rplA* | | | 24.69 | | | | | | C | | 2 | | | 13.9 | |  |  |  |  |  |
| lysyl-tRNA synthetase | | | | | | *lysS* | | | 56.68 | | | | | | C | | 2 | | | 4.85 | |  |  |  |  |  |
| DNA gyrase, A subunit | | | | | | *gyrA* | | | 99.32 | | | | | | C | | 2 | | | 2.48 | |  |  |  |  |  |
| staphylococcal accessory regulator S | | | | | | *sarS* | | | 29.87 | | | | | | C | | 2 | | | 8 | |  |  |  |  |  |
| 2,3-bisphosphoglycerate-independent phosphoglycerate mutase | | | | | | *gpmI* | | | 56.39 | | | | | | C | | 2 | | | 8.51 | |  |  |  |  |  |
| Dihydroorotase | | | | | | *pyrC* | | | 46.34 | | | | | | C | | 2 | | | 4.48 | |  |  |  |  |  |
| Isoleucyl-tRNA synthetase, mupirocin resistant protein | | | | | | *ileS* | | | 118.92 | | | | | | C | | 2 | | | 2.18 | |  |  |  |  |  |
| lipoteichoic acid synthase | | | | | | *ltaS* | | | 74.35 | | | | | | CM | | 41 | | | 56.04 | |  |  |  |  |  |
| ABC transporter, substrate-binding protein | | | | | | *mntC* | | | 34.72 | | | | | | CM | | 23 | | | 54.05 | |  |  |  |  |  |
| transferrin receptor | | | | | | *ceuA* | | | 37.83 | | | | | | CM | | 17 | | | 43.57 | |  |  |  |  |  |
| foldase protein PrsA precursor | | | | | | *prsA* | | | 35.62 | | | | | | CM | | 17 | | | 37.19 | |  |  |  |  |  |
| penicillin-binding protein 3 | | | | | | *pbp3* | | | 77.2 | | | | | | CM | | 17 | | | 29.23 | |  |  |  |  |  |
| iron compound ABC transporter, iron compound-binding protein SirA | | | | | | *sirA* | | | 36.72 | | | | | | CM | | 16 | | | 46.67 | |  |  |  |  |  |
| Lipoprotein | | | | | | SAUSA300_0437 | | | 30.44 | | | | | | CM | | 15 | | | 65.71 | |  |  |  |  |  |
| iron compound ABC transporter, iron compound-binding protein | | | | | | *fhuD2* | | | 33.99 | | | | | | CM | | 13 | | | 52.65 | |  |  |  |  |  |
| attachment of anionic polymers to PGN | | | | | | *lcpB* | | | 45.66 | | | | | | CM | | 13 | | | 39.51 | |  |  |  |  |  |
| methionine ABC transporter substrate-binding protein | | | | | | SAUSA300_0798 | | | 30.33 | | | | | | CM | | 13 | | | 54.95 | |  |  |  |  |  |
| Zn-binding lipoprotein adcA-like protein | | | | | | SAUSA300_2351 | | | 59.15 | | | | | | CM | | 11 | | | 24.42 | |  |  |  |  |  |
| penicillin-binding protein 2a | | | | | | *mecA* | | | 76.06 | | | | | | CM | | 10 | | | 18.71 | |  |  |  |  |  |
| ATP synthase F1, beta subunit | | | | | | *atpD* | | | 51.37 | | | | | | CM | | 8 | | | 22.98 | |  |  |  |  |  |
| Ferrichrome ABC transporter lipoprotein | | | | | | SAUSA300_2136 | | | 36.57 | | | | | | CM | | 7 | | | 24.46 | |  |  |  |  |  |
| MAP domain-containing protein | | | | | | *map* | | | 41.98 | | | | | | CM | | 7 | | | 14.63 | |  |  |  |  |  |
| quinol oxidase, subunit II | | | | | | *qoxA* | | | 41.75 | | | | | | CM | | 5 | | | 13.39 | |  |  |  |  |  |
| carboxyl-terminal protease | | | | | | *ctpA* | | | 55.23 | | | | | | CM | | 4 | | | 8.87 | |  |  |  |  |  |
| large conductance mechanosensitive channel protein | | | | | | *mscL* | | | 16.58 | | | | | | CM | | 3 | | | 25 | |  |  |  |  |  |
| peptide ABC transporter, peptide-binding protein | | | | | | SAUSA300_0073 | | | 57.91 | | | | | | CM | | 3 | | | 6.58 | |  |  |  |  |  |
| malate: quinone-oxidoreductase | | | | | | *mqo* | | | 56.98 | | | | | | CM | | 2 | | | 4.62 | |  |  |  |  |  |
| autolysin | | | | | | *atl* | | | 137.34 | | | | | | CW | | 48 | | | 46.97 | |  |  |  |  |  |
| sdrD protein | | | | | | *sdrD* | | | 149.36 | | | | | | CW | | 14 | | | 14.77 | |  |  |  |  |  |
| surface protein G | | | | | | *sasG* | | | 48.99 | | | | | | CW | | 13 | | | 38.51 | |  |  |  |  |  |
| secretory E matrix and plasma binding protein | | | | | | *emp* | | | 38.46 | | | | | | CW | | 7 | | | 28.82 | |  |  |  |  |  |
| oligopeptide permease, peptide-binding protein | | | | | | *opp-1A* | | | 59.98 | | | | | | CW | | 6 | | | 12.97 | |  |  |  |  |  |
| CHAP domain family autolysin | | | | | | *sle1* | | | 35.81 | | | | | | CW | | 6 | | | 25.75 | |  |  |  |  |  |
| sdrE protein | | | | | | *sdrE* | | | 125.19 | | | | | | CW | | 6 | | | 10.49 | |  |  |  |  |  |
| Clumping factor B | | | | | | *clfB* | | | 95.73 | | | | | | CW | | 6 | | | 7.79 | |  |  |  |  |  |
| Truncated FmtB protein | | | | | | *fmtB* | | | 117.24 | | | | | | CW | | 6 | | | 8.53 | |  |  |  |  |  |
| 5'-nucleotidase family protein | | | | | | SAUSA300_0025 | | | 83.37 | | | | | | CW | | 5 | | | 8.29 | |  |  |  |  |  |
| Clumping factor A | | | | | | *clfA* | | | 96.94 | | | | | | CW | | 5 | | | 6.43 | |  |  |  |  |  |
| E matrix-binding protein ebh | | | | | | *ebh* | | | 1121.91 | | | | | | CW | | 4 | | | 0.51 | |  |  |  |  |  |
| Iron-regulated surface determinant A | | | | | | *isdA* | | | 38.72 | | | | | | CW | | 3 | | | 11.14 | |  |  |  |  |  |
| Iron-regulated surface determinant B | | | | | | *isdB* | | | 72.15 | | | | | | CW | | 2 | | | 3.88 | |  |  |  |  |  |
| signal peptidase IB | | | | | | *spsB* | | | 17.59 | | | | | | CW | | 2 | | | 13.55 | |  |  |  |  |  |
| CHAP domain family protein | | | | | | SAUSA300_0651 | | | 28.17 | | | | | | CW | | 2 | | | 10.94 | |  |  |  |  |  |
| leukocidin B | | | | | | *lukB* | | | 38.66 | | | | | | E | | 38 | | | 68.93 | |  |  |  |  |  |
| triacylglycerol lipase precursor | | | | | | SAUSA300_0320 | | | 76.37 | | | | | | E | | 29 | | | 37.68 | |  |  |  |  |  |
| leukocidin A | | | | | | *lukA* | | | 40.41 | | | | | | E | | 21 | | | 53.85 | |  |  |  |  |  |
| Thermonuclease | | | | | | *nuc* | | | 25.1 | | | | | | E | | 19 | | | 53.51 | |  |  |  |  |  |
| IgG-binding protein Sbi | | | | | | *sbi* | | | 50.04 | | | | | | E | | 18 | | | 36.7 | |  |  |  |  |  |
| Panton-Valentine leukocidin, LukF-PV | | | | | | *lukF-PV* | | | 36.94 | | | | | | E | | 16 | | | 41.23 | |  |  |  |  |  |
| lantibiotic epidermin leader peptide processing serine protease EpiP | | | | | | epiP | | | 50.7 | | | | | | E | | 15 | | | 37.42 | |  |  |  |  |  |
| Panton-Valentine leukocidin, LukS-PV | | | | | | *lukS-PV* | | | 35.29 | | | | | | E | | 13 | | | 40.71 | |  |  |  |  |  |
| gamma-hemolysin component B | | | | | | *hlgB* | | | 36.69 | | | | | | E | | 13 | | | 43.69 | |  |  |  |  |  |
| leukotoxin LukD | | | | | | *lukD* | | | 36.87 | | | | | | E | | 11 | | | 32.72 | |  |  |  |  |  |
| N-acetylmuramoyl-L-alanine amidase domain protein | | | | | | SAUSA300_2579 | | | 69.18 | | | | | | E | | 10 | | | 27.79 | |  |  |  |  |  |
| staphopain A | | | | | | SAUSA300_1890 | | | 44.23 | | | | | | E | | 10 | | | 23.45 | |  |  |  |  |  |
| CamS sex pheromone cAM373 precursor | | | | | | SAUSA300_1884 | | | 45.35 | | | | | | E | | 9 | | | 31 | |  |  |  |  |  |
| leukotoxin LukE | | | | | *lukE* | | | | 34.8 | | | | E | | | | | | 9 | | 27.33 | |  |  |  |  |
| serine protease SplB | | | | | *splB* | | | | 26.08 | | | | | | | E | | | 8 | | | 31.67 | | |  |  |
| alpha-hemolysin | | | | | *hla* | | | | 35.95 | | | | | | | E | | | 7 | | | 29.47 | | |  |  |
| 1-phosphatidylinositol phosphodiesterase | | | | | *plc* | | | | 37.06 | | | | | | | E | | | 7 | | | 25 | | |  |  |
| gamma-hemolysin component C | | | | | *hlgC* | | | | 35.59 | | | | | | | E | | | 6 | | | 18.73 | | |  |  |
| staphylococcal enterotoxin-like toxin X | | | | | *selx* | | | | 23.15 | | | | | | | E | | | 6 | | | 29.56 | | |  |  |
| immunodominant antigen B | | | | | *isaB* | | | | 19.36 | | | | | | | E | | | 5 | | | 28.57 | | |  |  |
| cysteine protease precursor | | | | | *sspB* | | | | 44.49 | | | | | | | E | | | 4 | | | 7.89 | | |  |  |
| secretory antigen precursor SsaA | | | | | *ssaA* | | | | 29.31 | | | | | | | E | | | 3 | | | 13.48 | | |  |  |
| fibrinogen-binding protein | | | | | SAUSA300_1052 | | | | 12.59 | | | | | | | E | | | 3 | | | 25.69 | | |  |  |
| Superoxide dismutase (Mn/Fe family) | | | | | *SodA* | | | | 22.7 | | | | | | | E | | | 2 | | | 17.09 | | |  |  |
| fibrinogen-binding protein | | | | | *efb* | | | | 18.75 | | | | | | | E | | | 2 | | | 11.52 | | |  |  |
| Phenol-soluble modulin alpha 4 peptide | | | | | *psmA4* | | | | 2.17 | | | | | | | E | | | 2 | | | 85 | | |  |  |
| Phenol-soluble modulin alpha 3 peptide^e^ | | | | | *psmA3* | | | | 2.61 | | | | | | | E | | | 1 | | | 36.36 | | |  |  |
| phage portal protein | | | | | SAUSA300_1940 | | | | 45.49 | | | | | | | U | | | | 23 | | 65.06 | |  |  |  |
| amino acid ABC transporter, amino acid-binding protein | | | | | SAUSA300_2359 | | | | 28.89 | | | | | | | U | | | | 20 | | 63.32 | |  |  |  |
| glycerophosphoryl diester phosphodiesterase | | | | | *glpQ* | | | | 35.29 | | | | | | | U | | | | 20 | | 57.93 | |  |  |  |
| phi77 ORF006-like protein, putative capsid protein | | | | | SAUSA300_1938 | | | | 42.21 | | | | | | | U | | | | 19 | | 49.34 | |  |  |  |
| DNA-binding protein | | | | | SAUSA300_0146 | | | | | 56.27 | | | | | | U | | | | | | 12 | 34.24 | | | |
| putative lipoprotein | | | | | SAUSA300_0203 | | | | | 66.97 | | | | | | U | | | | | | 11 | 18.61 | | | |
| 3-methyl-2-oxobutanoate hydroxymethyltransferase | | | | | *panB* | | | | | 29.24 | | | | | | U | | | | | | 11 | 63.6 | | | |
| putative lipoprotein | | | | | SAUSA300_0769 | | | | | 28.4 | | | | | | U | | | | | | 9 | 39.26 | | | |
| putative lipoprotein | | | | | SAUSA300_0693 | | | | | 16.04 | | | | | | U | | | | | | 9 | 30.04 | | | |
| Transketolase | | | | | *tkt* | | | | | 68.32 | | | | | | U | | | | | | 8 | 19.04 | | | |
| conserved hypothetical protein | | | | | SAUSA300_0602 | | | | | 18.58 | | | | | | U | | | | | | 7 | 36.9 | | | |
| molybdenum ABC transporter, molybdenum-binding protein | | | | | *modA* | | | | | 29.03 | | | | | | U | | | | | | 6 | 28.08 | | | |
| staphylococcal tandem lipoprotein | | | | | SAUSA300_0419 | | | | | 31.42 | | | | | | U | | | | | | 6 | 22.96 | | | |
| 5'-nucleotidase, lipoprotein e(P4) family | | | | | SAUSA300_0307 | | | | | 33.33 | | | | | | U | | | | | | 6 | 28.38 | | | |
| 2,3-bisphosphoglycerate-dependent phosphoglycerate mutase | | | | | *gpmA* | | | | | 26.66 | | | | | | U | | | | | | 4 | 28.07 | | | |
| 50S ribosomal protein L21 | | | | | *rplU* | | | | | 11.33 | | | | | | U | | | | | | 4 | 43.14 | | | |
| putative lipoprotein | | | | | SAUSA300_0377 | | | | | 23.65 | | | | | | U | | | | | | 4 | 16.35 | | | |
| putative lipoprotein | | | | | SAUSA300_1478 | | | | | 14.09 | | | | | | U | | | | | | 4 | 39.83 | | | |
| chemotaxis-inhibiting protein CHIPS | | | | | *chp* | | | | | 17.05 | | | | | | U | | | | | | 3 | 22.82 | | | |
| ATP synthase F1, gamma subunit | | | | | *atpG* | | | | | 32.09 | | | | | | U | | | | | | 3 | 15.62 | | | |
| putative surface protein | | | | | SAUSA300_0408 | | | | | 56.51 | | | | | | U | | | | | | 2 | 6.81 | | | |
| putative helicase | | | | | SAUSA300_2431 | | | | | 109.83 | | | | | | U | | | | | | 2 | 1.78 | | | |
| conserved hypothetical protein | | | | | SAUSA300_2257 | | | | | 17.47 | | | | | | U | | | | | | 2 | 22.93 | | | |
| LtrC-like protein | | | | | pUSA030031 | | | | | 66.24 | | | | | | U | | | | | | 2 | 3.83 | | | |
| conserved hypothetical protein | | | | | *yloV* | | | | | 60.48 | | | | | | U | | | | | | 2 | 4.56 | | | |
| phi77 ORF020-like protein, phage major tail protein | | | | | SAUSA300_1934 | | | | | 23.93 | | | | | | U | | | | | | 2 | 11.21 | | | |
| putative lipoprotein | | | | | SAUSA300_0372 | | | | | 21.29 | | | | | | U | | | | | | 2 | 14.74 | | | |

**^a^** The proteins that were identified in JE2 WT EVs, but not in EVs from JE2*∆agr∆spa* mutant, are shown in bold type

**^b^** The predicted localization of proteins. C, Cytoplasmic; CM, Cytoplasmic membrane; CW, Cell wall; E, Extracellular; U, Unknown

**^c^** The number of peptide sequences that are unique to an identified protein

**^d^** The percentage of the protein sequence covered by identified peptides

**^e^** This PSMα3 peptide was identified with one unique peptide but protein coverage of 36.36%
