# Supplementary material for: Release of Staphylococcus aureus extracellular vesicles and their application as a vaccine platform
Source: Nat Commun. 2018 Apr 11;9:1379. doi: 10.1038/s41467-018-03847-z (PMC5895597; doi:10.1038/s41467-018-03847-z)
Supplement: Supplementary file 4 — Supplementary Data 2 [file 41467_2018_3847_MOESM4_ESM.docx]

**Release of *Staphylococcus aureus* extracellular vesicles and their application as a vaccine platform**

Wang et al.

**Supplementary Data 2 Proteins that were identified by LC-MS/MS in EVs purified from JE2∆*agr∆spa* mutant**

| Protein^a^ | | | Gene name | | | MW (kDa) | | | Predicted localization^b^ | | | Unique peptides^c^ | | | Coverage (%)^d^ | | | |  |  |
| --- | --- | --- | --- | --- | --- | --- | --- | --- | --- | --- | --- | --- | --- | --- | --- | --- | --- | --- | --- | --- |
| DNA-directed RNA polymerase, beta' subunit | | | *rpoC* | | | 135.2 | | | C | | | 32 | | | | 30.16 | | |  |  |
| DNA-directed RNA polymerase, beta subunit | | | *rpoB* | | | 133.14 | | | C | | | 25 | | | | 24.85 | | |  |  |
| dihydrolipoamide acetyltransferase | | | *pdhC* | | | 46.35 | | | C | | | 27 | | | | 65.95 | | |  |  |
| DNA polymerase I | | | *polA* | | | 99.13 | | | C | | | 34 | | | | 44.18 | | |  |  |
| 2-oxoisovalerate dehydrogenase, E3 component, lipoamide dehydrogenase | | | *lpdA* | | | 49.41 | | | C | | | 30 | | | | 63.25 | | |  |  |
| pyruvate dehydrogenase E1 component, alpha subunit | | | *pdhA* | | | 41.36 | | | C | | | 27 | | | | 65.95 | | |  |  |
| polyribonucleotide nucleotidyltransferase | | | *pnp* | | | 77.31 | | | C | | | 36 | | | | 58.6 | | |  |  |
| primosomal protein N` | | | *priA* | | | 92.46 | | | C | | | 25 | | | | 39.15 | | |  |  |
| pyruvate dehydrogenase E1 component, beta subunit | | | *pdhB* | | | 35.22 | | | C | | | 24 | | | | 74.77 | | |  |  |
| exonuclease RexA | | | *addA* | | | 141.2 | | | C | | | 19 | | | | 16.43 | | |  |  |
| type I restriction-modification enzyme, R subunit | | | *hsdR* | | | 109.16 | | | C | | | 18 | | | | 20.56 | | |  |  |
| phosphopyruvate hydratase | | | *eno* | | | 47.09 | | | C | | | 18 | | | | 49.77 | | |  |  |
| glucose-6-phosphate isomerase | | | *pgi* | | | 49.78 | | | C | | | 18 | | | | 36.34 | | |  |  |
| staphylococcal accessory regulator S | | | *sarS* | | | 29.87 | | | C | | | 15 | | | | 42.8 | | |  |  |
| exonuclease RexB | | | *addB* | | | 134.42 | | | C | | | 15 | | | | 14.42 | | |  |  |
| Catalase | | | *katA,* | | | 58.34 | | | C | | | 13 | | | | 32.48 | | |  |  |
| Protein translocase subunit SecA 1 | | | *secA1* | | | 95.9 | | | C | | | 13 | | | | 19.81 | | |  |  |
| Putative formate dehydrogenase | | | SAUSA300_2258 | | | 111.17 | | | C | | | 12 | | | | 16.16 | | |  |  |
| glycosyl transferase, group 1 family protein | | | SAUSA300_0550 | | | 58.38 | | | C | | | 12 | | | | 27.62 | | |  |  |
| ATP synthase F1, alpha subunit | | | *atpA* | | | 54.55 | | | C | | | 12 | | | | 26.29 | | |  |  |
| Ribonuclease J2 | | | *rnj2* | | | 62.56 | | | C | | | 12 | | | | 25.67 | | |  |  |
| ribosomal protein L1 | | | *rplA* | | | 24.69 | | | C | | | 12 | | | | 51.3 | | |  |  |
| ABC transporter substrate-binding protein | | | SAUSA300_0598 | | | 31.07 | | | C | | | 12 | | | | 52.73 | | |  |  |
| 30S ribosomal protein S2 | | | *rpsB* | | | 29.08 | | | C | | | 11 | | | | 35.69 | | |  |  |
| translation elongation factor Tu | | | *tuf* | | | 43.08 | | | C | | | 11 | | | | 45.18 | | |  |  |
| glutamyl-aminopeptidase | | | SAUSA300_2400 | | | 39.17 | | | C | | | 11 | | | | 43.58 | | |  |  |
| Ribonuclease J 1 | | | *rnj1* | | | 62.63 | | | C | | | 10 | | | | 22.83 | | |  |  |
| endonuclease IV | | | *nfo* | | | 33.14 | | | C | | | 10 | | | | 36.15 | | |  |  |
| cytosol aminopeptidase | | | *ampA* | | | 54.09 | | | C | | | 9 | | | | 26.07 | | |  |  |
| glyceraldehyde-3-phosphate dehydrogenase, type I | | | *gap* | | | 36.26 | | | C | | | 9 | | | | 33.04 | | |  |  |
| methionine--tRNA ligase | | | *metG* | | | 74.83 | | | C | | | 9 | | | | 16.44 | | |  |  |
| Alanine dehydrogenase 2 | | | *ald2* | | | 40.08 | | | C | | | 9 | | | | 32.26 | | |  |  |
| Dihydrolipoyllysine-residue succinyltransferase component of 2-oxoglutarate dehydrogenase complex | | | *odhB* | | | 46.64 | | | C | | | 9 | | | | 21.56 | | |  |  |
| DNA-directed RNA polymerase alpha subunit | | | *rpoA* | | | 34.99 | | | C | | | 9 | | | | 38.54 | | |  |  |
| L-lactate dehydrogenase 1 | | | | *ldh1* | | | 34.56 | | | C | | | 8 | | | | 25.87 | | |  |
| transcriptional regulator, gntR family protein | | | | SAUSA300_0503 | | | 53.92 | | | C | | | 8 | | | | 19.57 | | |  |
| cell division protein ftsA | | | | *ftsA* | | | 52.9 | | | C | | | 8 | | | | 18.3 | | |  |
| cell division protein FtsH | | | | *ftsH* | | | 77.74 | | | C | | | 7 | | | | 16.21 | | |  |
| DNA binding protein | | | | SAUSA300_2101 | | | 44.68 | | | C | | | 7 | | | | 19.05 | | |  |
| glycosyl transferase, group 1 family protein | | | | SAUSA300_0549 | | | 57.16 | | | C | | | 7 | | | | 18.78 | | |  |
| 50S ribosomal protein L2 | | | | *rplB* | | | 30.14 | | | C | | | 7 | | | | 36.46 | | |  |
| glutamine synthetase, type I | | | | *glnA* | | | 50.81 | | | C | | | 7 | | | | 20.85 | | |  |
| putative transcriptional regulator | | | | SAUSA300_2563 | | | 17.68 | | | C | | | 7 | | | | 37.09 | | |  |
| glycosyl transferase, group 1 family protein | | | | SAUSA300_0939 | | | 57.24 | | | C | | | 7 | | | | 15.21 | | |  |
| L-lactate dehydrogenase 2 | | | | *ldh2* | | | 34.4 | | | C | | | 7 | | | | 24.14 | | |  |
| purine nucleoside phosphorylase | | | | *deoD* | | | 25.89 | | | C | | | 6 | | | | 32.2 | | |  |
| fructose bisphosphate aldolase | | | | *fba* | | | 30.82 | | | C | | | 6 | | | | 28.67 | | |  |
| triosephosphate isomerase | | | | *tpiA* | | | 27.24 | | | C | | | 6 | | | | 23.32 | | |  |
| conserved hypothetical protein | | | | SAUSA300_1533 | | | 35.16 | | | C | | | 6 | | | | 20.67 | | |  |
| conserved hypothetical protein | | | | SAUSA300_1792 | | | 114.35 | | | C | | | 6 | | | | 8.28 | | |  |
| putative restriction/modification systems specificity protein | | | | SAUSA300_0406 | | | 47.11 | | | C | | | 6 | | | | 18.61 | | |  |
| dihydroorotase | | | | | *pyrC* | | | 46.34 | | | C | | | 6 | | | | 17.92 | | |
| pyruvate kinase | | | | | *pyk* | | | 63.06 | | | C | | | 6 | | | | 14.53 | | |
| Aerobic glycerol-3-phosphate dehydrogenase | | | | | *glpD* | | | 62.35 | | | C | | | 6 | | | | 13.29 | | |
| pyruvate carboxylase | | | | | *pyc* | | | 128.44 | | | C | | | 6 | | | | 5.74 | | |
| transcriptional regulator TcaR | | | | | *tcaR* | | | 17.51 | | | C | | | 5 | | | | 41.06 | | |
| HTH-type transcriptional regulator SarR | | | | | *sarR* | | | 13.66 | | | C | | | 5 | | | | 40 | | |
| staphylococcal accessory regulator A | | | | | *sarA* | | | 14.71 | | | C | | | 5 | | | | 33.06 | | |
| MraZ protein | | | | | *mraZ* | | | 17.23 | | | C | | | 5 | | | | 28.67 | | |
| ferritin-like protein | | | | | *ftnA* | | | 19.58 | | | C | | | 5 | | | | 27.11 | | |
| putative glutamyl aminopeptidase | | | | | SAUSA300_1261 | | | 37.83 | | | C | | | 5 | | | | 19.83 | | |
| 2-oxoisovalerate dehydrogenase, E2 component, dihydrolipoamide acetyltransferase | | | | | SAUSA300_1464 | | | 46.71 | | | C | | | 5 | | | | 12.5 | | |
| DNA gyrase, A subunit | | | | | *gyrA* | | | 99.32 | | | C | | | 5 | | | | 9.81 | | |
| riboflavin synthase, beta subunit | | | | | *ribH* | | | 16.39 | | | C | | | 4 | | | | 35.71 | | |
| staphylococcal accessory regulator V | | | | | *sarV* | | | 13.98 | | | C | | | 4 | | | | 33.62 | | |
| SpoVG protein | | | | | *spovG* | | | 11.27 | | | C | | | 4 | | | | 32 | | |
| phi77 ORF011-like protein, phage transcriptional repressor | | | | | SAUSA300_1969 | | | 27 | | | C | | | 4 | | | | 26.58 | | |
| ATP-dependent Clp protease | | | | | *clpP* | | | 21.5 | | | C | | | 4 | | | | 21.03 | | |
| D-alanine-activating enzyme/D-alanine-D-alanyl, dltD protein | | | | | *dltD* | | | 44.93 | | | C | | | 4 | | | | 14.58 | | |
| RNA binding protein CvfB | | | | | *cvfB* | | | 34.18 | | | C | | | 4 | | | | 14 | | |
| oligoendopeptidase F | | | | | *pepF* | | | 69.78 | | | C | | | 4 | | | | 10.93 | | |
| chaperone protein DnaK | | | | | *dnaK* | | | 66.32 | | | C | | | 4 | | | | 9.67 | | |
| 6-phosphogluconate dehydrogenase, decarboxylating | | | | | *gnd* | | | 51.77 | | | C | | | 4 | | | | 9.19 | | |
| glycosyl transferase, group 2 family protein | | | | SAUSA300_0252 | | | 66.27 | | | C | | | 4 | | | | 7.14 | | |  |
| DNA topoisomerase IV, subunit A | | | | *parC* | | | 91.02 | | | C | | | 4 | | | | 7.12 | | |  |
| DNA-binding protein HU | | | | *hup* | | | 9.62 | | | C | | | 3 | | | | 52.22 | | |  |
| general stress protein 20U | | | | *dps* | | | 16.68 | | | C | | | 3 | | | | 36.05 | | |  |
| 50S ribosomal protein L5 | | | | *rplE* | | | 20.25 | | | C | | | 3 | | | | 24.58 | | |  |
| monooxygenase family protein | | | | SAUSA300_2255 | | | 41.86 | | | C | | | 3 | | | | 10.43 | | |  |
| Undecaprenyl-diphosphatase | | | | *uppP* | | | 32.25 | | | C | | | 3 | | | | 8.93 | | |  |
| cell division protein ftsZ | | | | *ftsZ* | | | 41.01 | | | C | | | 3 | | | | 8.72 | | |  |
| Putative aldehyde dehydrogenase AldA | | | | *aldA* | | | 53.63 | | | C | | | 3 | | | | 6.87 | | |  |
| translation elongation factor G | | | | *fusA* | | | 76.56 | | | C | | | 3 | | | | 6.2 | | |  |
| 2-oxoglutarate dehydrogenase E1 component | | | | *odhA* | | | 105.28 | | | C | | | 3 | | | | 5.69 | | |  |
| aconitate hydratase | | | | *acnA* | | | 98.91 | | | C | | | 3 | | | | 5.33 | | |  |
| alanyl-tRNA synthetase | | | | *alaS* | | | 98.46 | | | C | | | 3 | | | | 4.68 | | |  |
| succinate dehydrogenase, flavoprotein subunit | | | | *sdhA* | | | 65.46 | | | C | | | 3 | | | | 4.93 | | |  |
| carbamoyl-phosphate synthase, large subunit | | | | *carB* | | | 117.11 | | | C | | | 3 | | | | 2.93 | | |  |
| transcription-repair coupling factor | | | | *mfD* | | | 134.14 | | | C | | | 3 | | | | 2.83 | | |  |
| putative arsenate reductase | | | | SAUSA300_0790 | | | 13.59 | | | C | | | 2 | | | | 31.36 | | |  |
| 30S ribosomal protein S7 | | | | *rpsG* | | | 17.78 | | | C | | | 2 | | | | 21.15 | | |  |
| conserved hypothetical protein | | | | SAUSA300_1797 | | | 17.79 | | | C | | | 2 | | | | 20.78 | | |  |
| conserved hypothetical protein | | | | SAUSA300_1863 | | | 10.32 | | | C | | | 2 | | | | 18.68 | | |  |
| 50S ribosomal protein L19 | | | | *rplS* | | | 13.35 | | | C | | | 2 | | | | 18.1 | | |  |
| conserved hypothetical protein | | | | SAUSA300_1685 | | | 17.99 | | | C | | | 2 | | | | 17.79 | | |  |
| 50S ribosomal protein L20 | | | | *rplT* | | | 13.68 | | | C | | | 2 | | | | 16.1 | | |  |
| 30S ribosomal protein S5 | | | | *rpsE* | | | 17.73 | | | C | | | 2 | | | | 15.66 | | |  |
| 30S ribosomal protein S8 | | | | *rpsH* | | | 14.82 | | | C | | | 2 | | | | 15.15 | | |  |
| acetyl-CoA carboxylase, biotin carboxyl carrier protein | | | | *accB* | | | 17.11 | | | C | | | 2 | | | | 12.99 | | |  |
| putative chromosome partitioning  protein, ParB family | | | | SAUSA300_2643 | | | 32.18 | | | C | | | 2 | | | | 12.54 | | |  |
| conserved hypothetical protein | | | | SAUSA300_1006 | | | 24 | | | C | | | 2 | | | | 11.27 | | |  |
| conserved hypothetical protein | | | | *murQ* | | | 32.36 | | | C | | | 2 | | | | 10.03 | | |  |
| glutamyl-aminopeptidase | | | | SAUSA300_1691 | | | 39.76 | | | C | | | 2 | | | | 9.78 | | |  |
| pyridoxine biosynthesis protein | | | | *pdxS* | | | 31.97 | | | C | | | 2 | | | | 9.49 | | |  |
| delta-aminolevulinic acid dehydratase | | | | *hemB* | | | 36.56 | | | C | | | 2 | | | | 8.95 | | |  |
| 50S ribosomal protein L22 | | | | *rplv* | | | 12.83 | | | C | | | 2 | | | | 7.69 | | |  |
| aldehyde dehydrogenase | | | | *aldA2* | | | 51.71 | | | C | | | 2 | | | | 7.63 | | |  |
| phosphoglycerate kinase | | | | *pgk* | | | 42.58 | | | C | | | 2 | | | | 7.32 | | |  |
| NAD-specific glutamate dehydrogenase | | | | *gudB* | | | 45.73 | | | C | | | 2 | | | | 6.52 | | |  |
| 6-phospho-beta-galactosidase | | | | *lacG* | | | 54.52 | | | C | | | 2 | | | | 6.38 | | |  |
| 2,3-bisphosphoglycerate-independent phosphoglycerate mutase | | | | *gpmI* | | | 56.39 | | | C | | | 2 | | | | 5.35 | | |  |
| methicillin resistance protein FemA | | | | *femA* | | | 50.62 | | | C | | | 2 | | | | 4.85 | | |  |
| isoleucyl-tRNA synthetase | | | | *ileS* | | | 104.82 | | | C | | | 2 | | | | 2.29 | | |  |
| DEAD-box ATP-dependent RNA helicase CshA | | | | *cshA* | | | 56.91 | | | C | | | 2 | | | | 3.75 | | |  |
| penicillin-binding protein 2a | | | | *mecA* | | | 76.06 | | | CM | | | 28 | | | | 43.71 | | |  |
| ABC transporter, substrate-binding protein | | | | *mntC* | | | 34.72 | | | CM | | | 25 | | | | 63.43 | | |  |
| penicillin binding protein 2 | | | | *pbp2* | | | 80.38 | | | CM | | | 24 | | | | 40.03 | | |  |
| Zn-binding lipoprotein adcA-like protein | | | | SAUSA300_2351 | | | 59.15 | | | CM | | | 19 | | | | 36.24 | | |  |
| MAP domain-containing protein, partial | | | | *map* | | | 41.98 | | | CM | | | 19 | | | | 32.45 | | |  |
| transferrin receptor | | | | SAUSA300_0721 | | | 37.83 | | | CM | | | 18 | | | | 46.2 | | |  |
| iron compound ABC transporter, iron compound-binding protein | | | | *fhuD2* | | | 33.99 | | | CM | | | 16 | | | | 58.61 | | |  |
| foldase protein PrsA precursor | | | | *prsA* | | | 35.62 | | | CM | | | 14 | | | | 33.44 | | |  |
| ATP synthase F1, beta subunit | | | | *atpD* | | | 51.37 | | | CM | | | 14 | | | | 38.51 | | |  |
| **Multifunctional fusion protein** | | | | *secD* | | | 84.2 | | | CM | | | 13 | | | | 20.42 | | |  |
| surface-associated protease HtrA | | | | SAUSA300_1674 | | | 45.78 | | | CM | | | 11 | | | | 26.18 | | |  |
| iron compound ABC transporter, iron compound-binding protein | | | | SAUSA300_2136 | | | 36.57 | | | CM | | | 10 | | | | 32.11 | | |  |
| oligopeptide permease, peptide-binding protein | | | | *opp-1A* | | | 59.98 | | | CM | | | 10 | | | | 22.74 | | |  |
| methionine ABC transporter substrate-binding protein | | | | SAUSA300_0798 | | | 30.33 | | | CM | | | 9 | | | | 35.16 | | |  |
| iron compound ABC transporter SirA | | | | *sirA* | | | 36.72 | | | CM | | | 9 | | | | 31.21 | | |  |
| Lipoprotein | | | | SAUSA300_0437 | | | 30.44 | | | CM | | | 9 | | | | 42.86 | | |  |
| quinol oxidase, subunit II | | | | *qoxA* | | | 41.75 | | | CM | | | 9 | | | | 24.59 | | |  |
| Malate dehydrogenase | | | | *mqo* | | | 56.98 | | | CM | | | 9 | | | | 22.89 | | |  |
| lipoteichoic acid synthase | | | | *ltaS* | | | 74.35 | | | CM | | | 8 | | | | 15.94 | | |  |
| phosphotransferase system, glucose-specific IIABC component | | | | *ptsG* | | | 73.88 | | | CM | | | 6 | | | | 14.24 | | |  |
| fructose specific permease | | | | *fruA* | | | 68.67 | | | CM | | | 6 | | | | 9.36 | | |  |
| septation ring formation regulator EzrA | | | | *ezrA* | | | 66.16 | | | CM | | | 6 | | | | 13.12 | | |  |
| AcrB/AcrD/AcrF family protein | | | | SAUSA300_2213 | | | 114.63 | | | CM | | | 6 | | | | 6.92 | | |  |
| conserved hypothetical protein | | | | SAUSA300_0844 | | | 44.08 | | | CM | | | 6 | | | | 18.91 | | |  |
| carboxyl-terminal protease | | | | *ctpA* | | | 55.23 | | | CM | | | 5 | | | | 13.51 | | |  |
| sortase A | | | | *srtA* | | | 23.53 | | | CM | | | 4 | | | | 25.24 | | |  |
| Lantibiotic transport ATP-binding protein | | | | *ecsA_1* | | | 25.77 | | | CM | | | 4 | | | | 17.75 | | |  |
| ABC transporter, ATP-binding protein | | | | *ybhF_1* | | | 32.95 | | | CM | | | 4 | | | | 17.93 | | |  |
| Serine/threonine protein kinase | | | | *pknB* | | | 74.32 | | | CM | | | 4 | | | | 6.33 | | |  |
| quinol oxidase, subunit I | | | | *qoxB* | | | 75.19 | | | CM | | | 4 | | | | 8.31 | | |  |
| putative lipoprotein | | | | SAUSA300_2315 | | | 23.35 | | | CM | | | 3 | | | | 26.32 | | |  |
| signal peptidase IB | | | | *spsB* | | | 17.59 | | | CM | | | 3 | | | | 21.29 | | |  |
| ABC transporter, ATP-binding protein | | | | SAUSA300_2357 | | | 27.22 | | | CM | | | 3 | | | | 19.34 | | |  |
| Putative lipoprotein | | | | SAUSA300_2354 | | | 23.05 | | | CM | | | 3 | | | | 18.09 | | |  |
| attachment of anionic polymers to PGN | | | | *lcpB* | | | 45.66 | | | CM | | | 3 | | | | 11.36 | | |  |
| penicillin-binding protein 4 | | | | *pbp4* | | | 48.23 | | | CM | | | 3 | | | | 9.74 | | |  |
| elastin-binding protein EbpS | | | | *ebpS* | | | 53.19 | | | CM | | | 2 | | | | 8.44 | | |  |
| ABC transporter ATP-binding protein | | | | SAUSA300_0620 | | | 28.01 | | | CM | | | 2 | | | | 8.5 | | |  |
| L-lactate permease | | | | *lctP* | | | 56.15 | | | CM | | | 2 | | | | 7.92 | | |  |
| pyruvate oxidase | | | | *cidC* | | | 63.72 | | | CM | | | 3 | | | | 7.77 | | |  |
| penicillin-binding protein 3 | | | | *pbp3* | | | 77.2 | | | CM | | | 3 | | | | 3.91 | | |  |
| amino acid carrier protein | | | | SAUSA300_1252 | | | 52.11 | | | CM | | | 2 | | | | 7.2 | | |  |
| peptide methionine sulfoxide reductase regulator MsrR | | | | *msrR* | | | 36.95 | | | CM | | | 2 | | | | 6.12 | | |  |
| Sodium:dicarboxylate symporter family protein | | | | *tcyP* | | | 49.4 | | | CM | | | 2 | | | | 6.93 | | |  |
| peptide ABC transporter peptide-binding protein | | | | SAUSA300_0073 | | | 57.91 | | | CM | | | 2 | | | | 5.42 | | |  |
| Autolysin | | | | *atl* | | | 137.34 | | | CW | | | 29 | | | | 34 | | |  |
| sdrD protein | | | | *sdrD* | | | 149.36 | | | CW | | | 25 | | | | 25.56 | | |  |
| Clumping factor B | | | | *clfB* | | | 95.73 | | | CW | | | 12 | | | | 17.13 | | |  |
| secretory E matrix and plasma binding protein | | | | *emp* | | | 38.46 | | | CW | | | 11 | | | | 36.76 | | |  |
| surface protein G | | | | *sasG* | | | 48.99 | | | CW | | | 10 | | | | 31.98 | | |  |
| SdrE protein | | | | *sdrE* | | | 125.19 | | | CW | | | 6 | | | | 7.8 | | |  |
| Clumping factor A | | | | *clfA* | | | 96.94 | | | CW | | | 5 | | | | 5.68 | | |  |
| truncated FmtB protein | | | | SAUSA300_2110 | | | 117.24 | | | CW | | | 3 | | | | 3.18 | | |  |
| truncated FmtB protein | | | | SAUSA300_2109 | | | 137.11 | | | CW | | | 3 | | | | 2.94 | | |  |
| IgG-binding protein Sbi | | | | *sbi* | | | 50.04 | | | E | | | 36 | | | | 66.28 | | |  |
| triacylglycerol lipase precursor | | | | SAUSA300_0320 | | | 76.37 | | | E | | | 22 | | | | 41.59 | | |  |
| secretory antigen precursor SsaA | | | | *ssaA* | | | 29.31 | | | E | | | 6 | | | | 35.58 | | |  |
| N-acetylmuramoyl-L-alanine amidase domain protein | | | | SAUSA300_2579 | | | 69.18 | | | E | | | 3 | | | | 7.11 | | |  |
| CamS sex pheromone cAM373 precursor | | | | SAUSA300_1884 | | | 45.35 | | | E | | | 3 | | | | 15.29 | | |  |
| LukA | | | | *lukA* | | | 40.41 | | | E | | | 3 | | | | 11.97 | | |  |
| LukB | | | | *luk*B | | | 38.66 | | | E | | | 3 | | | | 10.16 | | |  |
| amino acid ABC transporter, amino acid-binding protein | | | | SAUSA300_2359 | | | 28.89 | | | U | | | 20 | | | | 64.48 | | |  |
| phi77 ORF006-like protein, putative capsid protein | | | | SAUSA300_1938 | | | 42.21 | | | U | | | 17 | | | | 41.21 | | |  |
| molybdenum ABC transporter, molybdenum-binding protein ModA | | | | *modA* | | | 29.03 | | | U | | | 12 | | | | 43.46 | | |  |
| conserved hypothetical protein | | | | SAUSA300_0274 | | | 57.89 | | | U | | | 12 | | | | 24.46 | | |  |
| 3-methyl-2-oxobutanoate hydroxymethyltransferase | | | | *panB* | | | 29.24 | | | U | | | 11 | | | | 56.62 | | |  |
| type I restriction-modification system, M subunit | | | | *hsdM* | | | 59.41 | | | U | | | 11 | | | | 31.08 | | |  |
| conserved hypothetical protein | | | | SAUSA300_2394 | | | 23.79 | | | U | | | 10 | | | | 57.29 | | |  |
| putative lipoprotein | | | | SAUSA300_0693 | | | 16.04 | | | U | | | 8 | | | | 32.19 | | |  |
| conserved hypothetical protein | | | | SAUSA300_0317 | | | 30.24 | | | U | | | 7 | | | | 20.68 | | |  |
| conserved hypothetical protein | | | | SAUSA300_0602 | | | 18.58 | | | U | | | 6 | | | | 44.05 | | |  |
| putative lipoprotein | | | | SAUSA300_0372 | | | 21.29 | | | U | | | 6 | | | | 30.53 | | |  |
| putative lipoprotein | | | | SAUSA300_0377 | | | 23.65 | | | U | | | 6 | | | | 30.29 | | |  |
| 5'-nucleotidase, lipoprotein e(P4) family | | | | SAUSA300_0307 | | | 33.33 | | | U | | | 6 | | | | 28.38 | | |  |
| staphylococcal accessory protein X | | | | *sarX* | | | 14.17 | | | U | | | 4 | | | | 41.18 | | |  |
| HTH-transcriptional regulator rot | | | | *rot* | | | 18 | | | U | | | 4 | | | | 29.41 | | |  |
| putative lipoprotein | | | | SAUSA300_2403 | | | 17.3 | | | U | | | 4 | | | | 24.5 | | |  |
| putative lipoprotein | | | | SAUSA300_0992 | | | 23.86 | | | U | | | 4 | | | | 22.6 | | |  |
| type I restriction-modification enzyme, S subunit | | | | *hsdS* | | | 46.44 | | | U | | | 4 | | | | 17.79 | | |  |
| conserved hypothetical protein | | | | SAUSA300_1698 | | | 15.72 | | | U | | | 3 | | | | 32.14 | | |  |
| 2,3-bisphosphoglycerate-dependent phosphoglycerate mutase | | | | *gpmA* | | | 26.66 | | | U | | | 3 | | | | 16.23 | | |  |
| Fructose-bisphosphate aldolase class 1 | | | | *fda* | | | 33.03 | | | U | | | 3 | | | | 13.85 | | |  |
| phage portal protein | | | | SAUSA300_1940 | | | 45.49 | | | U | | | 3 | | | | 9.11 | | |  |
| 50S ribosomal protein L21 | | | | *rplU* | | | 11.33 | | | U | | | 2 | | | | 23.53 | | |  |
| 50S ribosomal protein L15 | | | | *rplO* | | | 15.59 | | | U | | | 2 | | | | 20.55 | | |  |
| putative lipoprotein | | | | SAUSA300_0769 | | | 28.4 | | | U | | | 2 | | | | 11.16 | | |  |
| putative lipoprotein | | | | SAUSA300_0079 | | | 20.12 | | | U | | | 2 | | | | 11.05 | | |  |
| staphylococcal tandem lipoprotein | | | | SAUSA300_0419 | | | 31.42 | | | U | | | 2 | | | | 10.37 | | |  |
| conserved hypothetical protein | | | | SAUSA300_2378 | | | 25.78 | | | U | | | 2 | | | | 9.59 | | |  |
| conserved hypothetical protein | | | SAUSA300_0198 | | | 35.53 | | | U | | | 2 | | | | 9.45 | | |  |  |
| conserved hypothetical protein | | | SAUSA300_1729 | | | 35.03 | | | U | | | 2 | | | | 8.94 | | |  |  |
| Transketolase | | | *tkt* | | | 68.32 | | | U | | | 2 | | | | 4.48 | | |  |  |

**^a^** The proteins that were identified in EVs from JE2∆*agr∆spa*, but not in JE2 WT EVs, are shown in bold type

**^b^** The predicted localization of proteins. C, Cytoplasmic; CM, Cytoplasmic membrane; CW, Cell wall; E, Extracellular; U, Unknown

**^c^** The number of peptide sequences that are unique to an identified protein

^d^ The percentage of the protein sequence covered by identified peptides
